# Robust immune responses are observed after one dose of BNT162b2 mRNA vaccine dose in SARS-CoV-2 experienced individuals

**DOI:** 10.1126/scitranslmed.abi8961

**Published:** 2021-12-07

**Authors:** Marie I. Samanovic, Amber R. Cornelius, Sophie L. Gray-Gaillard, Joseph Richard Allen, Trishala Karmacharya, Jimmy P. Wilson, Sara Wesley Hyman, Michael Tuen, Sergei B. Koralov, Mark J. Mulligan, Ramin Sedaghat Herati

**Affiliations:** ^1^ NYU Langone Vaccine Center, Department of Medicine, New York University Grossman School of Medicine; New York, NY 10016, USA.; ^2^ Department of Pathology, New York University School of Medicine; New York, NY 10016, USA.

## Abstract

The use of COVID-19 vaccines will play the major role in helping to end the pandemic that has killed millions worldwide. COVID-19 vaccines have resulted in robust humoral responses and protective efficacy in human trials, but efficacy trials excluded individuals with a prior diagnosis of COVID-19. As a result, little is known about how immune responses induced by mRNA vaccines differ in individuals who recovered from COVID-19. Here, we evaluated longitudinal immune responses to two-dose BNT162b2 mRNA vaccination in 15 adults who had experienced COVID-19, compared to 21 adults who did not have prior COVID-19. Consistent with prior studies of mRNA vaccines, we observed robust cytotoxic CD8^+^ T cell responses in both cohorts following the second dose. Furthermore, SARS-CoV-2-naive individuals had progressive increases in humoral and antigen-specific antibody-secreting cell (ASC) responses following each dose of vaccine, whereas SARS-CoV-2-experienced individuals demonstrated strong humoral and antigen-specific ASC responses to the first dose but these responses were not further enhanced after the second dose of the vaccine at the time points studied. Together, these data highlight the relevance of immunological history for understanding vaccine immune responses and may have implications for personalizing mRNA vaccination regimens used to prevent COVID-19, including for the deployment of booster shots.

## Teaser

Prior history of COVID-19 enhances adaptive immune responses to mRNA vaccination.

## INTRODUCTION

Severe acute respiratory syndrome coronavirus 2 (SARS-CoV-2) has caused hundreds of millions of infections and millions of deaths worldwide (*1*). Although repeated infection has been described (*2*, *3*), resolution of SARS-CoV-2 infection was associated with reduced susceptibility to re-infection in animal models (*4*) and in humans (*5*). However, it remains unknown how long this protection lasts. A number of promising vaccine candidates have emerged, including mRNA vaccines, vector-based vaccines, and protein-adjuvant vaccines (*6*). Maintenance of protective immune responses by vaccines will be important for preventing de novo and recurrent infection with SARS-CoV-2.

Identification of protective correlates of immunity will be critical to predicting susceptibility to SARS-CoV-2 infection. Humoral responses have been identified as a correlate of immunity for a variety of pathogens (*7*). In the setting of SARS-CoV-2 infection in non-human primates, humoral responses conferred protection and T cell responses were partially protective in the setting of waning antibody titers (*8*). Indeed, studies with mRNA vaccine candidates against SARS-CoV-2 have induced robust humoral responses against SARS-CoV-2 in animal models (*9*–*11*) and in humans (*12*–*17*) and were efficacious in large-scale clinical trials (*18*, *19*). In addition to humoral responses, mRNA vaccines induced type 1 responses in CD4^+^ T cells, as evidenced by enzyme-linked immunospot (ELISpot) and intracellular cytokine staining for interferon (IFN)-γ and tumor necrosis factor (TNF) (*13*, *14*). Recent studies have highlighted antigen-specific B cell responses (*20*) and germinal center formation after mRNA vaccination (*21*). However, the full spectrum of immune responses to the vaccines has not been evaluated.

Memory is the hallmark of adaptive immune responses and typically results in faster response to the pathogen upon re-exposure. Immunological history can radically shape subsequent immune responses in other ways. For example, influenza susceptibility has been linked to strain-specific exposure from decades earlier (*22*, *23*). Moreover, non-neutralizing antibody responses to acute dengue infection are a risk factor for antibody-dependent disease enhancement for serodiscordant strains (*24*, *25*). These, and other examples from the literature (*26*), further highlight the importance for understanding immunological history in the context of coronavirus disease 2019 (COVID-19) vaccines. Moreover, large-scale clinical trials excluded individuals with a prior diagnosis of COVID-19, thereby leaving an unexplored gap in our understanding of vaccine responses in SARS-CoV-2-experienced individuals. Indeed, given the scope of the pandemic, addressing this gap in knowledge will be relevant to hundreds of millions of recovered individuals worldwide.

Here, our goal was to evaluate the effects of a prior history of COVID-19 on the immune response to mRNA vaccination. Following COVID-19, humoral and cellular immune responses persist (*27*–*29*), but little is known about the effects of prior COVID-19 on subsequent exposure to SARS-CoV-2 proteins. In an observational study, we longitudinally evaluated immune responses to mRNA vaccines in adults who were naive to SARS-CoV-2 (SARS-CoV-2-naive) or who had experienced SARS-CoV-2 infection (SARS-CoV-2-experienced). Using unbiased high-dimensional flow cytometry analyses, we observed cytotoxic CD8^+^ T cell responses to vaccination in both cohorts. However, further analysis revealed subtle differences between cohorts. We found evidence for altered SARS-CoV-2-specific antibody-secreting cell (ASC) induction in circulation and altered humoral responses to vaccination depending on prior history of COVID-19. Better understanding of how prior COVID-19 shapes the immune responses to COVID-19 vaccines will improve our ability to predict susceptibility and enable personalized vaccine strategies for maintenance of immunity with booster shots.

## RESULTS

### Robust T cell responses are observed following mRNA vaccination.

**Fig. 1.**
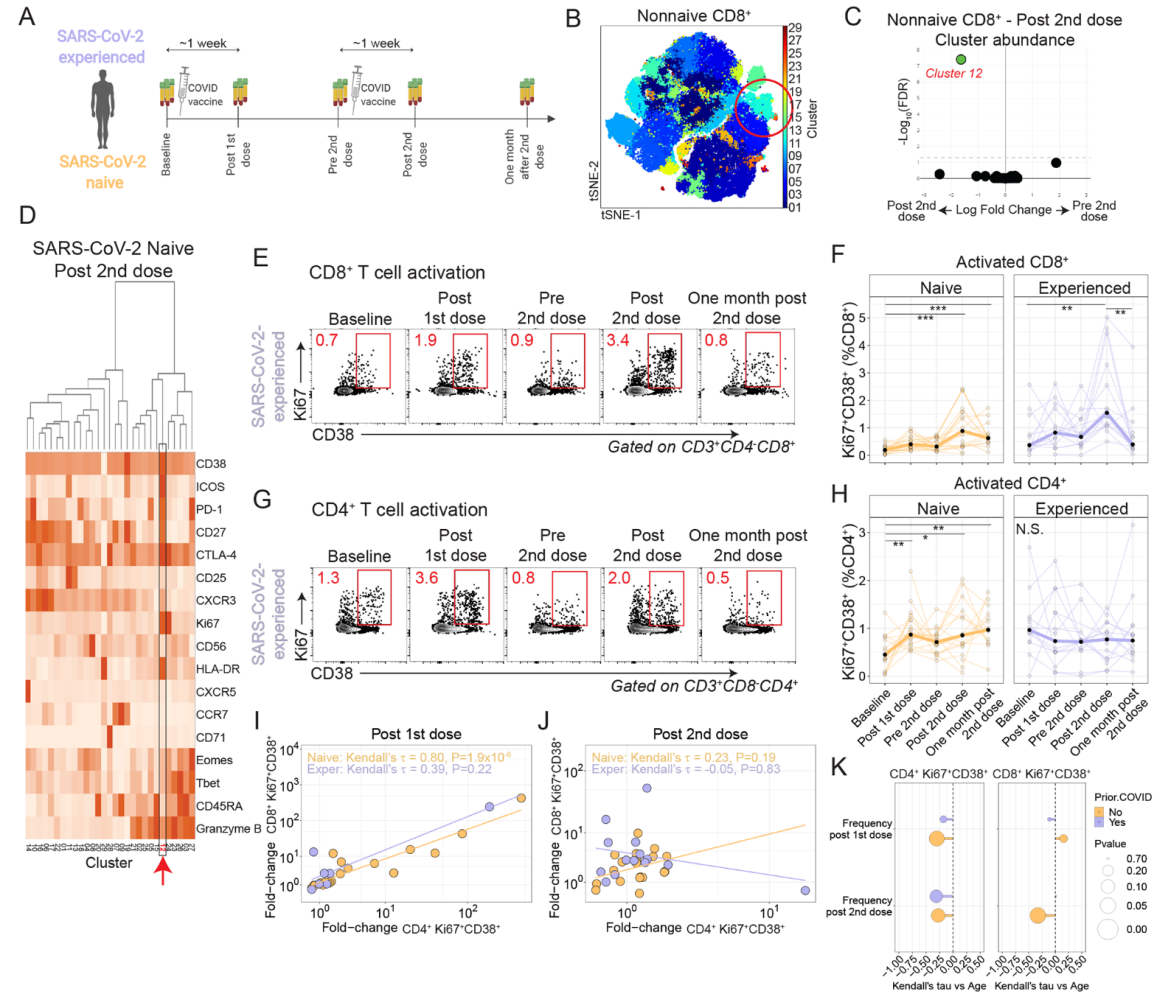
mRNA vaccination induces CD4^+^ and CD8^+^ T cell responses. (**A**) Study schematic. (**B**) Non-naive CD8^+^ T cells from all participants were colored using Phenograph clusters and projected using tSNE. The circled region indicates cluster 12. (**C**) Phenograph cluster abundance for non-naive CD8^+^ T cells was compared using edgeR for all participants before and after the second vaccination. (**D**) The heatmap shows non-naive CD8^+^ T cell cluster protein expression for SARS-CoV-2-naive participants after the second vaccination. The red arrow indicates cluster 12. (**E**) Example flow cytometry plots show CD8^+^ T cell expression of Ki67 and CD38 in an experienced participant. Red numbers indicate frequency. (**F**) Summary data for Ki67^+^CD38^+^ expression in CD8^+^ T cells is shown by cohort. **P < 0.01 and ***P < 0.001 by Dunn’s post-test for SARS-CoV-2-naive (orange, n=19) and SARS-CoV-2-experienced (purple, n=14) participants. Thick lines represent median of values. (**G**) Example flow cytometry plots show CD4^+^ T cell expression of Ki67 and CD38 in a SARS-CoV-2 experienced participant. Red numbers indicate frequency. (**H**) Summary data for Ki67^+^CD38^+^ expression in CD4^+^ T cells is shown by cohort. *P < 0.05, **P < 0.01, and ***P < 0.001 by Dunn’s post-test for SARS-CoV-2-naive (orange, n=19) and SARS-CoV-2-experienced (purple, n=14) participants. Thick lines represent median of values. (**I and J**) Kendall rank correlations are shown for the fold-changes were calculated for CD8^+^Ki67^+^CD38^+^ and CD4^+^Ki67^+^CD38^+^ T cells at one week after first dose compared to baseline (I) or at one week after second dose compared to Pre 2nd dose time point (J) for SARS-CoV-2-naive (orange, n=19) and SARS-CoV-2-experienced (purple, n=12) participants. (**K**) A Kendall correlation is shown for the comparison of CD4^+^Ki67^+^CD38^+^ subset versus age. Nominal *P*-values are shown for SARS-CoV-2-naive (orange, n=21) and SARS-CoV-2-experienced (purple, n=14) participants.

Prior immune history can affect subsequent responses to antigen (*30*). To test the effects of immunological history in the setting of COVID-19, we recruited 15 individuals who had laboratory-confirmed COVID-19 (hereafter labeled SARS-CoV-2-experienced) and 21 individuals who did not have documented COVID-19 (hereafter labeled SARS-CoV-2-naive). Participants’ ages ranged from 21 to 65, with a median age of 39 for naive adults and 43 for SARS-CoV-2-experienced individuals (table S1). All SARS-CoV-2-experienced adults had mild COVID-19 or asymptomatic infection except one individual who had severe disease (table S2). Two individuals were infected with SARS-CoV-2 within 30 days prior to vaccination, whereas the remaining 13 were at least eight months beyond diagnosis of COVID-19. For these two cohorts, all participants received two doses of the BNT162b2 mRNA vaccine in accordance with the FDA Emergency Use Authorization. Peripheral immune responses were assessed before and after each dose of vaccine (**Fig. 1A**). Relative to the start of vaccination, samples were categorized as Baseline, Post 1st dose (6 to 13 days after vaccination), Pre 2nd dose (immediately prior to second vaccination and about 21 days since initial vaccination), Post 2nd dose (7 to 12 days after second vaccination), and One month post 2nd dose (about 4 weeks after second vaccination) (**Fig. 1A** and fig. S1A). We opted to look at one week after second vaccination as that was the peak of the humoral response following mRNA vaccination (*12*, *16*, *31*).

To determine the phenotype of circulating T cells responding to vaccination, we performed high-dimensional spectral flow cytometry longitudinally for all participants (fig. S1B and table S3). We initially reasoned that T cell responses would be most evident following the second dose, thus we performed cluster analysis (*32*) and *t-*distributed stochastic neighbor embedding (tSNE) representation of all non-naive CD8^+^ T cells pooled from all time points and all participants (**Fig. 1B**). Of the 29 clusters identified, only a single cluster (Cluster 12) increased in abundance at the Post 2nd dose time point compared to Pre 2nd dose (**Fig. 1C** and fig. S1C). Cells in Cluster 12 expressed multiple proteins that are associated with activation, including Ki67, CD38, and Inducible costimulator (ICOS) (**Fig. 1D** and fig. S1D). We next assessed these cells longitudinally using manual gating for Ki67 and CD38. Indeed, we found that vaccination was associated with robust induction of Ki67^+^CD38^+^ CD8^+^ T cells one week after each vaccination (**Fig. 1, E** and **F**), which was consistent with prior reports of robust induction of cytotoxic T cells after vaccination (*33*). Compared to baseline Ki67^+^CD38^+^ CD8^+^ T cell frequencies, the first vaccination induced a median 1.9-fold increase for SARS-CoV-2-naive and for SARS-CoV-2-experienced individuals one week post first dose. However, compared with the Pre 2nd dose time point, the second vaccination induced a 2.2-fold and 3.3-fold increase in SARS-CoV-2-naive and -experienced individuals, respectively, at one week post second dose. We also considered whether there might be differential timing of CD8^+^ T cell responses between the two cohorts, but analysis of time as a continuous variable did not identify a consistent pattern (fig. S1, E and F). Moreover, Ki67^+^CD38^+^ CD8^+^ T cells expressed Granzyme B, suggesting strong cytotoxic potential, and responded with memory kinetics to repeat exposure to SARS-CoV-2 antigens (fig. S1, G and H). Together, these data show that mRNA vaccination was associated with cytotoxic CD8^+^ T cell responses in both cohorts.

We next asked if similar changes were evident in circulating CD4^+^ T cells. Here, cluster analysis of non-naive CD4^+^ T cells identified 22 clusters, two of which increased in abundance after the second dose of vaccine: Clusters 13 and 21 (fig. S1, I and J). Of these responding clusters, Cluster 13 was associated with high expression of Ki67, CD38, and ICOS (fig. S1, K and L). Indeed, longitudinal analysis revealed induction of Ki67^+^CD38^+^ CD4^+^ T cells following immunization in the SARS-CoV-2-naive adults, with a 1.9-fold increase after first vaccination compared to baseline and a 1.3-fold increase at Post 2nd dose compared to Pre 2nd dose time points (**Fig. 1, G and H**). In contrast, we observed muted CD4^+^ responses in SARS-CoV-2-experienced adults. We considered whether there might be differential timing of CD4^+^ T cell responses between the two cohorts but again did not identify a consistent pattern (fig. S1, M and N). Interestingly, the two participants with recent COVID-19 infection showed the strongest CD8^+^ T cell dynamic responses after each dose in the experienced cohort, whereas their CD4^+^ T cell response remained muted (fig. S1O). These data demonstrated induction of an activated CD4^+^ T cell population after vaccination in SARS-CoV-2-naive adults with only muted responses in SARS-CoV-2-experienced adults.

We next asked if these activated CD4^+^ and CD8^+^ T cell responses were correlated. Indeed, we found strong positive correlation between activated CD4^+^ and CD8^+^ responses after the first dose of vaccine and a weak correlation after the second dose of vaccine in SARS-CoV-2-naive adults (**Fig. 1, I and J**). In contrast, activated CD4^+^ and CD8^+^ responses in SARS-CoV-2-experienced adults had only a modest correlation after the first dose and no correlation after the second dose of vaccine. We also considered other demographic variables in the analysis. Aging has been associated with reduced vaccine immunogenicity and effectiveness (*34*). Indeed, COVID-19 mortality increases with age (*35*), and it remains unclear how well COVID-19 vaccines perform in older adults (*36*). Here, we observed no correlation with age in activated CD8^+^ T cell responses. In contrast, we found negative correlations for activated CD4^+^ T cell responses with participant age following both primary and second vaccinations (**Fig. 1K** and fig. S1, P and Q). These results indicated the potential for reduced CD4^+^ T cell responses to vaccination with aging and underscored the need for additional studies to fully understand the effects of aging on mRNA vaccine-induced immune responses.

### Antigen-specific T cell responses are induced by mRNA vaccination.

We observed T cell responses based on phenotypic changes in both cohorts (**Fig. 1****)**, which led to the hypothesis that the vaccination had induced antigen-specific T cell responses. We first considered cytokine production following stimulation, as prior studies demonstrated the presence of TNF- or IFN-γ-producing T cells following mRNA immunization (*14*). We performed overnight stimulation of peripheral blood mononuclear cells (PBMCs) with peptide pools for the SARS-CoV-2 spike protein across all time points, followed by intracellular staining for IFN-γ or TNF. Anti-spike protein T cell responses were observed at baseline (fig. S2, A to F), consistent with reports of pre-existing cross-reactivity (*37*). Following vaccination, we observed progressively higher frequencies of CD8^+^ and CD4^+^ T cells producing these cytokines in response to peptide stimulation among SARS-CoV-2-naive adults but stable frequencies of CD8^+^ and CD4^+^ T cells producing these cytokines among SARS-CoV-2-experienced adults. These data indicated that antigen-specific T cells were induced by vaccination in SARS-CoV-2-naive adults.

**Fig. 2.**
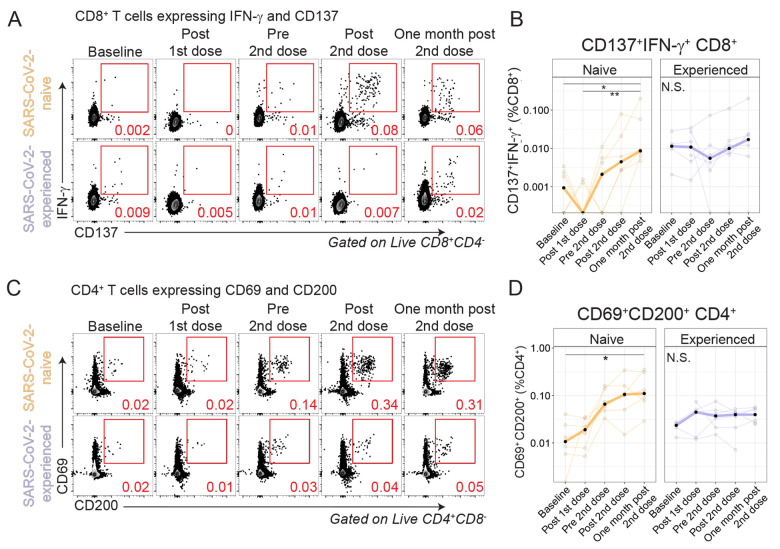
Antigen-specific T cells are induced by vaccination. PBMCs were rested overnight then stimulated for 20 hours with SARS-CoV-2 spike peptides in the presence of monensin, followed by phenotypic analysis. (**A**) Example flow cytometry plots are shown for CD8^+^ T cells isolated from SARS-CoV-2-naive and SARS-CoV-2-experienced individuals evaluated for the expression of IFN-γ and CD137 for the full time course. (**B**) Summary data of IFN-γ and CD137 expression are shown for SARS-CoV-2-naive (orange, n=6) and SARS-CoV-2-experienced (purple, n=6) participants. Thick lines represent median of values. (**C**) Example flow cytometry plots are shown for CD4^+^ T cells isolated from SARS-CoV-2-naive and SARS-CoV-2-experienced individuals evaluated for the expression of CD69 and CD200 for the full time course. (**D**) Summary data are shown for SARS-CoV-2-naive (orange, n=6) and SARS-CoV-2-experienced (purple, n=6) participants. Thick lines represent mean values. Red numbers in (A) and (C) indicate frequency. *P < 0.05 and **P < 0.01 by Kruskal-Wallis analysis and Dunn’s post-test.

We next considered other means of identifying antigen-specific cells. We (*38*) and others (*37*, *39*, *40*) have demonstrated the use of activation-induced markers (AIM) as a method to study antigen-specific T cells that may not produce cytokines. Following overnight stimulation of PBMCs with peptide pools for the SARS-CoV-2 spike protein, we identified induction of CD137^+^IFN-γ^+^ CD8^+^ cells after vaccination in SARS-CoV-2-naive adults but not in SARS-CoV-2-experienced adults (**Fig. 2, A and B**, and fig. S2G). We next considered CD4^+^ T cell induction by vaccination (**Fig. 2, C and D**, and fig. S2H). Similar to CD8^+^ T cell responses, CD4^+^ T cells coexpressing CD69 and CD200 after stimulation increased 9-fold at one month post second vaccination relative to baseline in SARS-CoV-2-naive adults (*P*=0.025, Kruskal-Wallis with Dunn’s post-test) but did not increase in SARS-CoV-2-experienced adults. We also considered other surface proteins including co-expression of OX40 and CD137 and observed similar results (fig. S2, I to L). In sum, spike protein-specific T cell responses were induced in SARS-CoV-2-naive adults but did not further increase in SARS-CoV-2-experienced adults after mRNA vaccination.

### Differential induction of circulating T follicular helper cells after vaccination was observed between those with and without prior history of COVID-19.

Most vaccines are thought to confer protection by induction of a class-switched, affinity-matured antibody response (*7*), and, given the subtle differences in CD4^+^ T cell responses following mRNA vaccination between cohorts (**Fig. 1**), we next considered CD4^+^ T cell responses that might be relevant to the antibody response. Maturation of B cell responses within germinal centers requires help from CD4^+^ T follicular cells (Tfh) (*41*, *42*). Indeed, spike protein-specific germinal center B cells were identified in axillary lymph node aspirates after mRNA vaccination (*21*). However, lymphoid tissue is challenging to routinely study in humans. We, and others, have focused on a circulating Tfh-like subset with similar phenotypic, transcriptional, epigenetic, and functional characteristics to lymphoid Tfh (*43*–*47*). Indeed, we previously found that vaccination induced antigen-specific ICOS^+^CD38^+^ circulating Tfh (cTfh) which correlated with plasmablast responses and demonstrated memory kinetics (*38*). Furthermore, other studies identified similar activated cTfh responses in non-human primates following mRNA vaccination for influenza (*48*). However, activated cTfh have not been evaluated in humans following SARS-CoV-2 mRNA vaccination.

**Fig. 3.**
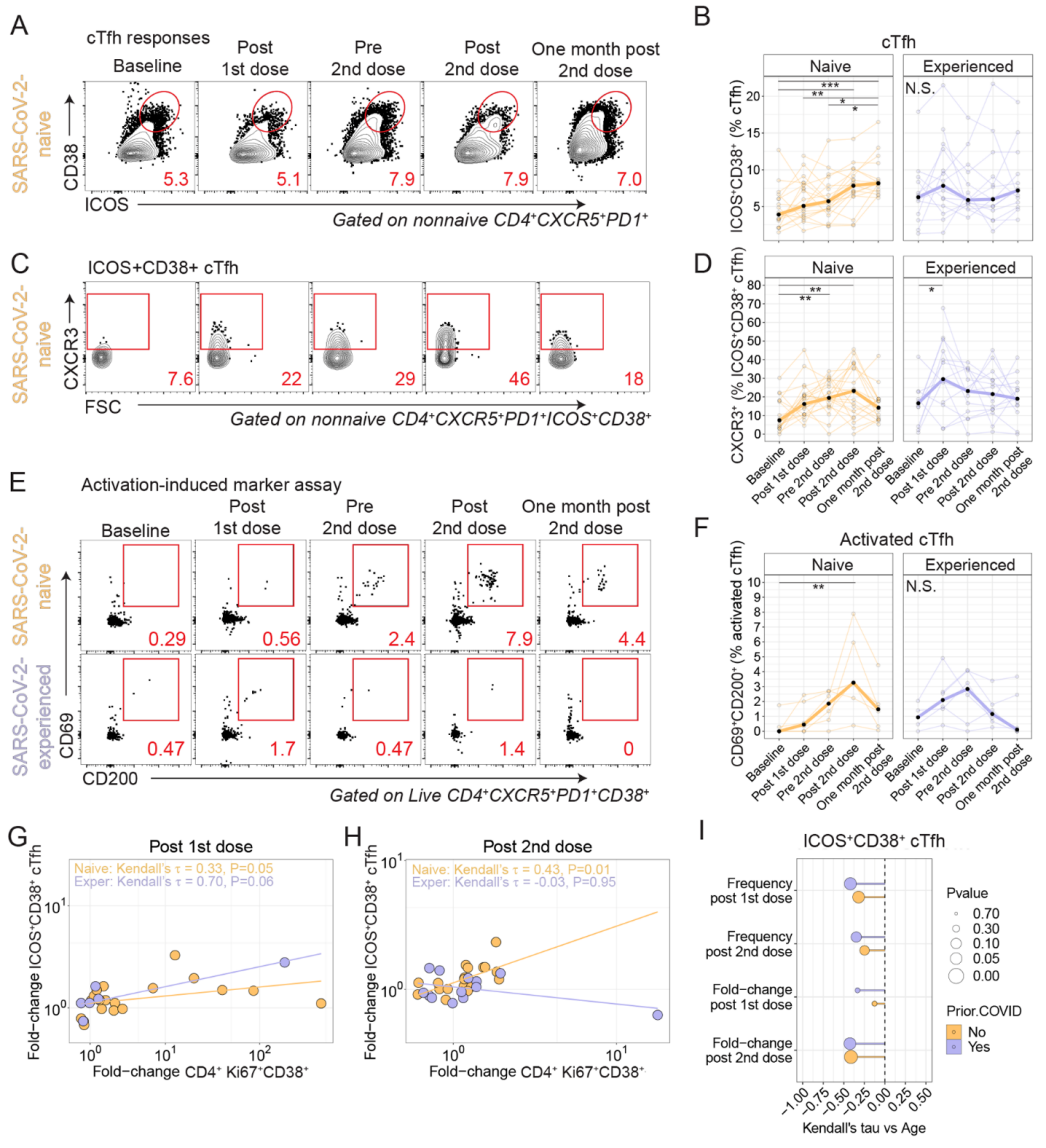
Circulating T follicular helper cells are differentially induced in SARS-CoV-2 naive and experienced individuals after vaccination. (**A**) Example flow cytometry plots are shown for a sample from a naive participant stained for ICOS and CD38 in cTfh. Red numbers indicate frequency. (**B**) Summary data are shown for expression of ICOS and CD38 in cTfh for SARS-CoV-2-naive (orange, n=21) and SARS-CoV-2-experienced (purple, n=14) participants. Thick lines represent median of values. (**C**) Example flow cytometry plots are shown for CXCR3 expression in ICOS^+^CD38^+^ cTfh. Red numbers indicate frequency. FSC, forward scatter. (**D**) Summary data for CXCR3 expression is shown for ICOS^+^CD38^+^ cTfh isolated from SARS-CoV-2-naive (orange, n=21) and SARS-CoV-2-experienced (purple, n=14) participants. Thick lines represent median of values. (**E**) PBMCs were stimulated with overlapping SARS-CoV-2 spike peptides for 20 hours followed by phenotypic analysis. CD38^hi^ cTfh were assessed for expression of CD69 and CD200. Red numbers indicate frequency. (**F**) Summary plots for CD69 and CD200 coexpression among CD38^hi^ cTfh are shown for SARS-CoV-2-naive (orange, n=6) and SARS-CoV-2-experienced (purple, n=6) participants. Thick lines represent median of values. (**G and H**) Kendall correlations are shown between the fold-change in ICOS^+^CD38^+^ cTfh and Ki67^+^CD38^+^ CD4^+^ T cells at one week Post 1st dose compared to baseline (G) or one week after second dose compared to Pre 2nd dose (H) for SARS-CoV-2-naive (orange, n=19) and SARS-CoV-2-experienced (purple, n=12) participants. (**I**) Kendall correlations are shown for the comparison of CD4^+^Ki67^+^CD38^+^ subset versus age. Fold-change Post 1st dose is relative to baseline, and fold-change Post 2nd dose is relative to Pre 2nd dose time point. Nominal *P*-values shown. For all plots, *P < 0.05, **P < 0.01, and ***P < 0.001 by Dunn’s post-test unless otherwise noted.

We scrutinized all time points for evidence of cTfh responses. ICOS^+^CD38^+^ cTfh cells increased following vaccination in SARS-CoV-2-naive adults and peaked one week after the second vaccine dose (**Fig. 3, A and B**). In contrast, SARS-CoV-2-experienced adults did not show similar induction of cTfh cells following either dose of the vaccine (**Fig. 3B**). In prior studies, antigen-specific ICOS^+^CD38^+^ cTfh expressed CXCR3 following influenza vaccination (*38*, *46*). Thus we next considered the subset of ICOS^+^CD38^+^ cTfh that expressed CXCR3. Here, we identified a 2.1-fold induction of CXCR3^+^ cells among ICOS^+^CD38^+^ cTfh cells in SARS-CoV-2-experienced adults after the first vaccine dose and a 2.0-fold increase among SARS-CoV-2-naive adults after the first dose (**Fig. 3, C and D**). There was minimal change in CXCR3 expression in ICOS^+^CD38^+^ cTfh one week after the second dose of vaccine in either cohort. Of note, SARS-CoV-2-experienced participants with recent COVID-19 showed a more dynamic induction of CXCR3^+^ cells among ICOS^+^CD38^+^ cTfh cells after each vaccine dose (fig. S3A).

We next used the AIM assay to identify antigen-specific cTfh induced by vaccination, as we have done previously (*38*). Spike protein-specific activated cTfh in the AIM assay were identified as CD4^+^ T cells expressing CXCR5 and programmed cell death protein 1 (PD-1) that had high expression of CD38 and that coexpressed CD69 and CD200 after overnight stimulation with a spike peptide pool (**Fig. 3, E and F**). In this subset, we found a 14.5-fold induction of cells that among SARS-CoV-2-naive adults at the Post 2nd dose time point relative to baseline (*P*=0.008, Kruskal-Wallis with Dunn’s post-test), whereas no increase was observed among SARS-CoV-2-experienced adults during the same time interval (**Fig. 3, E and F**), which was similar to the trend observed for the CXCR3^+^ICOS^+^CD38^+^ cTfh (**Fig. 3, C and D**). Together, these data demonstrated the induction of spike protein-specific cTfh in SARS-CoV-2-naive adults following mRNA vaccination.

Thus, we asked if the cTfh response correlated with the Ki67^+^CD38^+^ CD4^+^ response. Indeed, ICOS^+^CD38^+^ cTfh from SARS-CoV-2-naive adults correlated positively with Ki67^+^CD38^+^ CD4^+^ T cells for the fold-change at Post 1st dose compared to baseline (**Fig. 3G** and fig. S3B) and at Post 2nd dose compared to Pre 2nd dose (**Fig. 3H** and fig. S3C). In contrast, SARS-CoV-2-experienced adults had a positive correlation after the first dose and did not have a correlation after the second dose. We also found negative correlations with age in both cohorts (**Fig. 3I**), similar to what was observed for activated CD4^+^ responses (**Fig. 1**).

We also evaluated other well-established cellular correlates of the humoral response such as plasmablasts (*48*), CD21^lo^ B cells (*49*), and CD71^+^ B cells (*50*). However, we found little or no induction of these subsets in either cohort longitudinally (fig. S3, D to J). Plasma CXCL13, which has been reported as a plasma biomarker of early germinal center activity (*51*), also did not change following vaccination in either cohort (fig. S3, K and L).

Altogether, we found antigen-specific induction of ICOS^+^CD38^+^ cTfh following vaccination with subtle differences between cohorts. Indeed, although the ICOS^+^CD38^+^ cTfh frequency continued to increase in the SARS-CoV-2-naive adults there was no evidence of sustained induction of cTfh in SARS-CoV-2-experienced adults over the course of the vaccination series. Given that Tfh provide help to B cells, these data provoked the question as to whether B cell responses also differed by prior history of COVID-19.

### Fewer SARS-CoV-2-specific ASCs are present in circulation after two vaccine doses in SARS-CoV-2-experienced verus naïve adults.

We observed subtle differences in induction of ICOS^+^CD38^+^ cTfh following vaccination based on prior history of COVID-19 (**Fig. 3**). Thus, we next asked if antigen-specific B cell responses induced by vaccination were influenced by prior exposure to the virus. To test this, we performed ELISpot analyses of ASCs for reactivity against SARS-CoV-2 proteins one week after each vaccine dose for both cohorts.

**Fig. 4.**
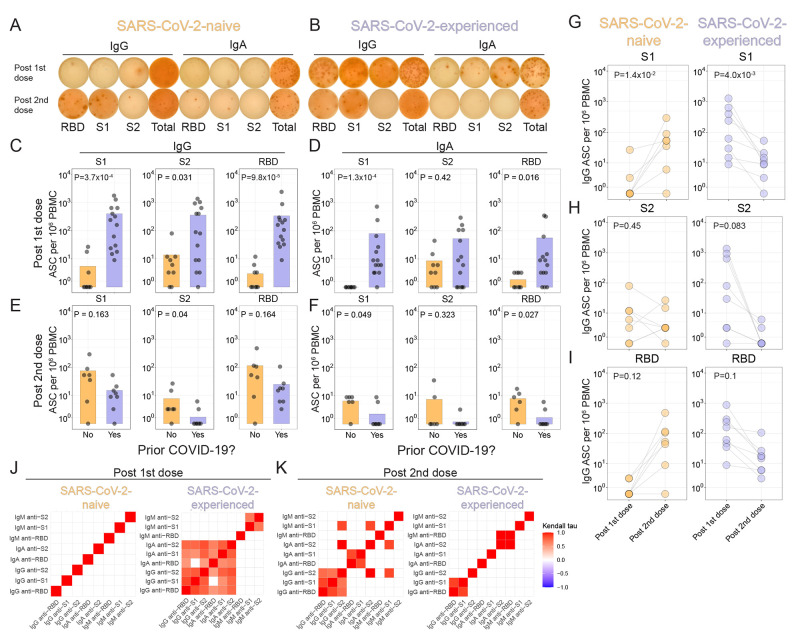
Few antigen-specific ASCs are induced in circulation after the second vaccine dose in SARS-CoV-2-experienced adults. (**A and B**) Example antibody-secreting cell (ASC) ELISpots for a SARS-CoV-2-naive (A) or SARS-CoV-2-experienced (B) adult one week after each dose of vaccine are shown. (**C to F**) Summary statistics for ELISpot assays are shown. For each panel, S1 (left), S2 (middle), or RBD (right) antigens for IgG or IgA are represented, at one week after first dose (C and D) for SARS-CoV-2-naive (orange, n=9) and SARS-CoV-2-experienced (purple, n=13) participants or one week after second dose (E and F) for SARS-CoV-2-naive (orange, n=6) and SARS-CoV-2-experienced (purple, n=8) participants. Nominal *P* values were calculated by Wilcoxon tests. (**G to I**) ELISpot results for SARS-CoV-2-naive (left, n=7) or SARS-CoV-2-experienced (right, n=8) adults are shown for S1 (**G**), S2 (**H**), or RBD (**I**). Connected lines indicate repeated measurements from the same participants. Nominal *P* values from paired *t* tests are presented. (**J and K**) Kendall correlations are shown for ELISpot results one week after the first vaccination (J) or one week after the second vaccination (K) for SARS-CoV-2-naive (n=9) and SARS-CoV-2-experienced (n=13) participants. Correlations shown for comparisons with nominal *P* values <0.05.

Given the persistence of SARS-CoV-2-reactive B cells in individuals who recovered from COVID-19 (*28*), we expected to find a stronger antigen-specific ASC response in SARS-CoV-2-experienced adults than SARS-CoV-2-naive adults after the first dose of vaccine. Indeed, after the first dose of vaccine, SARS-CoV-2-naive adults had few SARS-CoV-2-specific ASCs detected, whereas SARS-CoV-2-experienced adults had stronger IgG-secreting ASC responses to the receptor binding domain (RBD), S1 domain, and S2 domain (**Fig. 4, A** to **C****,** fig. S4, A and B). Moreover, IgA-secreting ASCs were identified predominantly in SARS-CoV-2-experienced adults after the first vaccine dose, albeit at a somewhat lower frequency than IgG-secreting ASCs (**Fig. 4D**). Few IgM-secreting ASC were identified (fig. S4C). Although global plasmablast frequencies did not change with vaccination (fig. S3, D to F), we did indeed find evidence of antigen-specific ASCs responses following the first vaccine dose among both cohorts.

We next asked if the second vaccination also induced strong antigen-specific ASC responses in the two cohorts. Indeed, the second dose of vaccine robustly induced S1- and RBD-reactive ASCs in SARS-CoV-2-naive adults (**Fig. 4, E to I** and fig. S4D). In contrast, however, the second dose of vaccine induced similar, or weaker, ASC responses in SARS-COV-2-experienced adults approximately one week after vaccination for all three SARS-CoV-2 antigens tested (**Fig. 4, E to I** and fig. S4D). Spike protein-specific ASC induction was correlated by isotype and antigen in SARS-CoV-2-experienced adults one week after the first vaccination and SARS-CoV-2-naive one week after the second vaccination (**Fig. 4, J and K**, fig. S4, E to H). However, correlations by isotype and antigen were not observed in the SARS-CoV-2-experienced adults following the second vaccination. Furthermore, the pattern of these correlations changed between doses. For example, among SARS-CoV-2-naive adults, there was correlation between IgM-, IgA-, and IgG-secreting ASCs after the second vaccination but not after first vaccination, whereas similar correlations were observed in ASCs from SARS-CoV-2-experienced adults after the first, but not after the second, vaccination. The lack of correlation may have been partly due to the low numbers of ASCs detected, but further studies will be needed to determine how unswitched and switched B cell populations respond in the settings of priming and recall responses. Together, these data demonstrated increased induction of antigen-specific ASC responses with repeated vaccination in SARS-CoV-2-naive adults, whereas fewer antigen-specific ASCs were observed in circulation with repeated vaccination in SARS-CoV-2-experienced adults.

### Antigen-specific B cells are induced by vaccination.

**Fig. 5.**
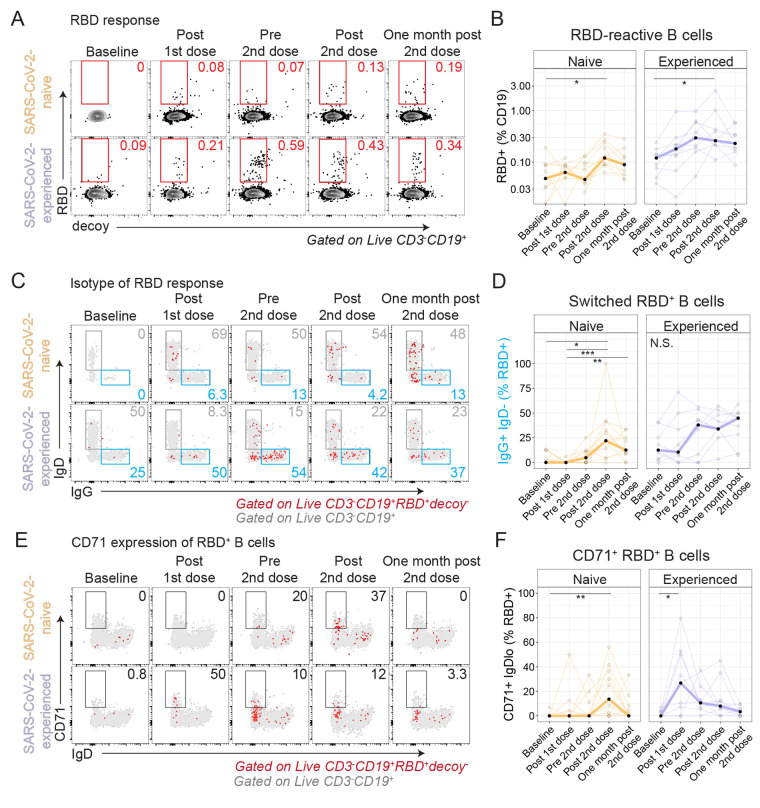
Antigen-specific B cells are induced by immunization. Recombinant biotinylated RBD was tetramerized and used to detect RBD-reactive B cells by flow cytometry. For all plots, *P < 0.05, **P < 0.01, and ***P < 0.001 by Dunn’s post-test. (**A and B**) Example flow cytometry gating for RBD^+^ decoy^-^ CD19^+^ B cells (A) and summary plots (B) are shown for SARS-CoV-2-naive (orange, n=11) and SARS-CoV-2-experienced (purple, n=9) participants. Red numbers indicate frequency. Thick lines represent median of values. (**C**) RBD^+^ B cell expression of IgD and IgG is shown, with RBD^+^ decoy^-^ B cells (red) overlaid on all CD19^+^ B cells (gray). Blue and gray numbers indicate frequency. (**D**) Summary plots for data presented in (C) are shown for SARS-CoV-2-naive (orange, n=11) and SARS-CoV-2-experienced (purple, n=9) participants. Thick lines represent median of values. (**E**) RBD^+^ B cell expression of IgD and CD71 is shown for RBD^+^ B cells (red) overlaid on all CD19^+^ B cells (gray). Black numbers indicate frequency. (**F**) Summary plots for data presented in (E) are shown for SARS-CoV-2-naive (orange, n=11) and SARS-CoV-2-experienced (purple, n=9) participants. Thick lines represent median of values.

To further study the induction of SARS-CoV-2-specific B cell responses after immunization, we used fluorescent recombinant RBD protein to identify RBD-reactive B cells in PBMCs (fig. S5A). We found RBD-reactive B cells were 2-fold higher at baseline among SARS-CoV-2-experienced adults compared to SARS-CoV-2-naive adults (*P*=0.03, Wilcoxon test) (fig. S5B). Furthermore, fewer RBD-reactive B cells were class-switched B cells in SARS-CoV-2-naive adults, as evidenced by expression of IgG, relative to SARS-CoV-2-experienced adults (*P*=0.02, Wilcoxon test, fig. S5C). Following immunization, increased frequencies of RBD^+^ B cells were observed in both cohorts (**Fig. 5, A and B**), with fold-changes of 2.9 and 4.8 in SARS-CoV-2-naive and SARS-CoV-2-experienced adults, respectively, at Post 2nd dose relative to Baseline. Moreover, SARS-CoV-2-naive adults had progressively more IgG^+^ RBD^+^ B cells at Post 2nd dose than at Baseline (*P*=0.01, Kruskal-Wallis with Dunn’s post-test, **Fig. 5, C and D**), whereas SARS-CoV-2-experienced adults had minimal change in the proportion of IgG^+^ RBD^+^ B cells at the same time points (*P*=0.24, one-way ANOVA with Tukey’s post-test).

We next considered plasmablast differentiation, which is likely to be necessary for durable humoral immunity. Indeed, plasma cells were identified in the bone marrow of adults following COVID-19 mRNA vaccination (*52*). Though we identified ASCs (**Fig. 4**), we did not identify RBD^+^ B cells that had a CD27^+^CD38^hi^ phenotype, presumably due to down-regulation of the B cell receptor. However, other B cell subsets relevant to plasmablast differentiation were identified. For example, CD71^+^ B cells, which were described following influenza vaccination (*50*), were induced most strongly by the second immunization in SARS-CoV-2-naive adults but were induced most strongly by the first immunization in SARS-CoV-2-experienced adults (**Fig. 5, E and F**, fig. S5D). Progressive B cell differentiation is associated with reduced expression of CD24 (*53*). Indeed, we found that some RBD^+^ B cells had low expression of CD24 in both cohorts after vaccination (fig. S5, E and F). Similarly, B cells with low expression of CD21, which are thought to be precursors to long-lived plasma cells (*49*), were induced, albeit weakly, in the RBD^+^ B cell subset after vaccination (fig. S5G). Double negative (DN) 2 B cells (*54*–*56*) were also increased in the RBD^+^ B cell subset after immunization (fig. S5, H and I). Each of these populations could represent different populations of mixed activated B cells and antibody secreting cells; nonetheless these data together indicated that RBD^+^ B cells were induced by immunization and likely progressively developed phenotypes associated with differentiation.

### Humoral responses differ by history of COVID-19.

**Fig. 6.**
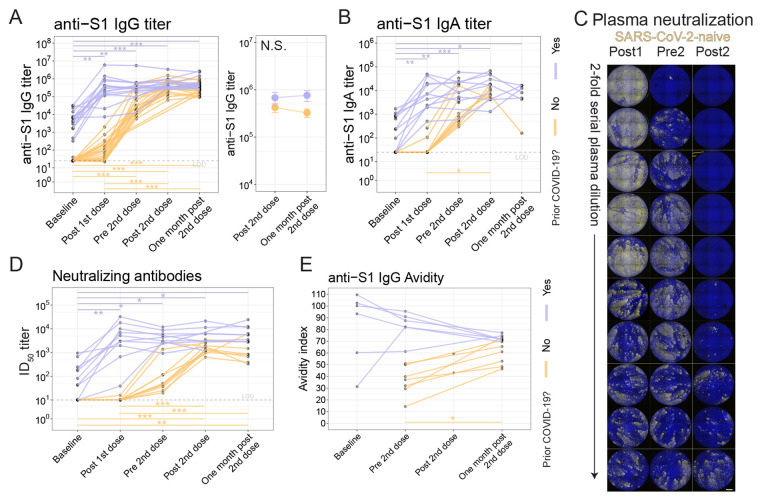
Antibody responses differ based on prior history of COVID-19. (**A**) Anti-S1 IgG antibody titers were assessed for SARS-CoV-2 naive (orange, n=21) and SARS-CoV-2-experienced (purple, n=15) adults (left) with connected lines indicating repeated measurements of the participants over time. At right, anti-S1 IgG antibody titer summarized at the Post 2nd dose and One month post 2nd dose time points for each cohort. Symbols indicate mean, and error bars indicate standard error. LOD indicates limit of detection. (**B**) Anti-S1 IgA antibody titers are shown for SARS-CoV-2-naive (orange, n=10) and SARS-CoV-2-experienced (purple, n=13) participants. (**C**) Plasma neutralizing antibody titers were assessed with an in vitro microneutralization assay using SARS-CoV-2 live virus. A representative serial dilution series is shown for one participant’s plasma at the Post 1st dose (Post1), Pre 2nd dose (Pre2), and Post 2nd dose (Post2) time points. Scale bar indicates 100 μm. (**D**) Neutralizing antibody titers are shown as log_10_ of half-maximal inhibitory dilution (ID_50_) for SARS-CoV-2-naive (orange, n=10) and SARS-CoV-2-experienced (purple, n=10) participants. (**E**) Anti-S1 IgG antibody avidity was assessed using a urea wash ELISA. Data are expressed as a ratio of urea washed-absorbance to unwashed absorbance for SARS-CoV-2-naive (orange, n=7) and SARS-CoV-2-experienced (purple, n=6) participants. For all plots, N.S., not significant, *P < 0.05, **P < 0.01, and ***P < 0.001 by Dunn’s post-test unless otherwise noted.

ASC induction differed by prior history of COVID-19 (**Fig. 4**), thus we next asked whether humoral responses were affected by prior history of COVID-19. To test this, we first assessed antibody responses to the S1 subunit of the spike protein (*57*). As previously demonstrated (*28*), anti-S1 IgG antibodies were detectable in individuals who had recovered from COVID-19 and were not detectable in those who were SARS-CoV-2-naive at baseline (median titers 5991 and 25, respectively; *P*=3.1x10^−8^; Wilcoxon test) (**Fig. 6A** and fig. S6A). Following first dose immunization, SARS-CoV-2-experienced adults had a median fold-change of 92 whereas SARS-CoV-2-naive adults had a median fold-change of only 2.7 (*P*=6.9x10^−4^; Wilcoxon test). However, after second dose immunization, SARS-CoV-2-experienced adults had a median fold-change of only 1.3 whereas the SARS-CoV-2-naive adults had a fold-change of 11 (*P*=3.3x10^−5^; Wilcoxon test). Compared to the SARS-CoV-2-naive cohort, the SARS-CoV-2-experienced cohort had similar anti-S1 IgG titers at the Post 2nd dose time point but had 1.75-fold higher titers at One month post 2nd dose (*P*=5.3x10^−3^; Wilcoxon test, fig. S6B). However, no difference was observed between the Post 2nd dose and One month post 2nd dose time points for either cohort (paired *t*-test, **Fig. 6A**). An overall similar pattern was observed for anti-S1 IgA titers (**Fig. 6B**), and, as expected, vaccination did not affect anti-nucleocapsid antibodies (fig. S6, C and D). The change in anti-S1 IgG titer in SARS-CoV-2-experienced adults was inversely correlated with their titer at baseline (fig. S6E). Thus, these data demonstrate rapid and robust humoral responses after initial vaccination in both cohorts but minimal further increase in SARS-CoV-2-experienced adults after the second vaccine dose.

In a subset of participants, we asked if live-virus neutralizing antibodies were induced following immunization. We observed low titers of neutralizing antibodies at baseline in SARS-CoV-2-experienced adults, whereas plasma from SARS-CoV-2-naive adults did not have detectable neutralizing antibodies (**Fig. 6, C and D**, fig. S6F). Following the first immunization, SARS-CoV-2-experienced adults had a rapid increase in neutralizing antibody titers to a median of 4084, whereas SARS-CoV-2-naive adults achieved a titer of 10 (*P*=8.7x10^−5^; Wilcoxon test). As observed earlier with anti-S1 binding antibodies, subsequent neutralizing antibody titers were largely unchanged by second vaccination in SARS-CoV-2-experienced adults, at least over the observed period, whereas the neutralizing titers in SARS-CoV-2-naive adults continued to increase. Nonetheless, neutralizing titers remained higher in the SARS-CoV-2-experienced adults compared to the SARS-CoV-2-naive adults at the Post 2nd dose time point (fig. S6G), and also at the One month post 2nd dose time point (*P*=2.9x10^−3^, Wilcoxon test) (fig. S6H). We also considered whether participants with a recent COVID-19 diagnosis had any effect on vaccine responses compared to participants with more distant COVID-19 diagnosis but did not find clear effects (fig. S6 I to K). In sum, we found that neutralizing antibody responses induced by SARS-CoV-2 mRNA vaccination in SARS-CoV-2-experienced adults were of greater magnitude than in naive adults.

Antibody avidity has been used to assess affinity maturation following vaccination (*58*–*60*). To assess avidity, urea wash enzyme-linked immunosorbent assays (ELISAs) was performed for anti-S1 IgG antibodies on serum samples longitudinally. In SARS-CoV-2-naive adults, avidity continued to increase steadily over the measured time points (**Fig. 6E**), including at One month post 2nd dose when antibody titers had plateaued. This prolonged period of increasing avidity is consistent with the recent report of human axillary lymph node germinal center reactions lasting at least 5 weeks post-RNA vaccination (*21*). All SARS-CoV-2-experienced adults assayed had relatively high-avidity antibodies at baseline, but, in contrast to SARS-CoV-2-naive adults, avidity decreased in four of five participants over time and with second vaccination, which may have been due to the induction of new, low avidity humoral responses that had not undergone germinal center maturation. All together, these data demonstrated pronounced differences in humoral responses based on prior history of COVID-19 (fig. S6L).

## DISCUSSION

Prior studies have demonstrated the importance of humoral and cellular responses for susceptibility to COVID-19 (*8*). Better understanding of factors that affect immune responses will be critical to the design of next generation COVID-19 vaccines and their optimal use, including booster shots. Here, we observed subtle differences in cellular responses and more pronounced differences in humoral responses between individuals naive to SARS-CoV-2 and those who had recovered from SARS-CoV-2 infection. Both cohorts had similar CD8^+^ T cell responses to vaccination, which were typified by co-expression of Ki67 and CD38. In contrast, antigen-specific humoral cell responses differed by cohort. SARS-CoV-2-experienced adults had more antigen-specific ASCs in circulation one week after the first vaccination compared to SARS-CoV-2-naive adults, but the frequency of antigen-specific ASCs after second vaccination did not increase in previously infected individuals, unlike the SARS-CoV-2-naive adults. In both cohorts, however, RBD-specific B cells increased in frequency in circulation following vaccination. Additionally, prior history of COVID-19 was associated with 100- to 1000-fold increase in anti-spike protein IgG antibody titers following the first vaccination, with limited increase upon the second vaccination; in contrast, antibody titers increased steadily over time in SARS-CoV-2-naive adults.

Prior studies demonstrated potent induction of T cell responses in animal models (*33*). Consistent with these studies, we also observed robust induction of cytotoxic CD8^+^ T cell responses following vaccination. The Ki67^+^CD38^+^ CD4^+^ T cell responses we observed were consistent with other reports that have identified antigen-specific CD4^+^ T cell responses following mRNA vaccination (*14*). Moreover, we demonstrated the induction of spike protein-specific responses in both CD8^+^ and CD4^+^ T cells among SARS-CoV-2-naive adults using AIM. Although a robust CD8^+^Ki67^+^CD38^+^ T cell response was observed phenotypically in PBMCs from SARS-CoV-2-experienced adults, the AIM responses were low in magnitude. Further optimization of the peptide pools may be needed, as other studies have observed strong peptide-specific CD8^+^ responses. Additional studies of antigen specificity, such as by T cell tetramer assays, may help resolve T cell responses to individual epitopes. Future studies will be needed to determine whether spike protein-specific T cell responses after mRNA immunization in adults are a correlate of protection.

Durable, affinity-matured antibody responses require germinal center reactions in lymphoid tissue (*42*), and germinal centers are established following mRNA vaccination (*21*). Consistent with this, our spike protein antibody avidity experiments demonstrated progressive maturation of serum antibodies following mRNA vaccination, with increasing avidity through at least a month post second vaccination. However, much remains to be learned about Tfh responses in this context and whether these cells play a role in antibody responses after mRNA vaccination (*61*). Two prior studies evaluated mRNA vaccination for influenza in humans and non-human primates and found robust induction of ICOS^+^CXCR3^+^PD-1^+^ cTfh responses and neutralizing antibodies (*48*, *62*). Here, CXCR3^+^ICOS^+^CD38^+^ cTfh were induced by vaccination in both cohorts. Furthermore, we previously demonstrated that antigen-specific cTfh responses were induced by influenza vaccination which correlated with cellular and humoral responses to vaccination (*38*, *63*). Here as well, we found that mRNA vaccination induced spike protein-specific activated cTfh. However, activated cTfh are, at best, a proxy for understanding lymphoid Tfh responses in humans (*43*, *63*). Plasma CXCL13, which increased after yellow fever vaccination, but not influenza vaccination (*51*, *64*), was not different following mRNA vaccination in our study, which may indicate relative differences in extent of germinal center formation or perhaps differences in early germinal center events that lead to CXCL13 production. Additional studies involving direct lymph node sampling, as was done recently (*65*, *66*), will be needed to understand Tfh dynamics and memory formation, and additional time points will be needed to determine the utility of cellular and humoral biomarkers, including plasma CXCL13 and the plasmablast response, following mRNA vaccination.

Notable qualitative and quantitative differences in immune responses were observed when comparing adults who were naive to SARS-CoV-2 to those who had recovered from SARS-CoV-2 infection. Anti-S1 binding IgG antibodies and neutralizing antibodies appeared to peak one week after second vaccination, consistent with published reports of humoral responses to mRNA vaccination (*12*, *18*). By the end of the observation period, SARS-CoV-2-experienced adults had higher titers of anti-S1 binding IgG and neutralizing antibodies at the One month post 2nd dose time point, compared to SARS-CoV-2-naive adults. Moreover, humoral responses continued to qualitatively change in avidity for spike protein, despite the plateau in antibody quantity. In SARS-CoV-2-naive adults, affinity increased over time, which may reflect germinal center-related affinity maturation (*11*, *67*). In contrast, SARS-CoV-2-experienced adults had reduction in affinity over time, presumably reflecting the contribution of de novo B cell responses that had not undergone affinity maturation, rather than loss of high-affinity antibodies. Indeed, better understanding the qualitative and quantitative changes in antibodies over time will have major implications for the need for booster vaccinations.

Furthermore, humoral responses were robust after the first vaccination but more muted after the second vaccination in SARS-CoV-2-experienced adults, and this pattern was also evident in anti-S1 IgG and anti-S1 IgA antibodies, as well as live-virus neutralizing antibodies. Several possibilities may explain these differences. For example, the early plateau in humoral responses could indicate altered B cell differentiation away from antigen-specific plasma cells, which would be consistent with the reduced ASC responses in SARS-CoV-2-experienced adults after the second dose. In addition, the reduction in antigen-specific ASCs may have also altered trafficking of ASCs following repeat vaccination, perhaps shifting the peak ASC response earlier than was assessed here. Another possibility is that the very high titers of anti-S1 IgG responses may restrict antigen availability for stimulation of non-memory B cell clones following subsequent vaccine doses. Indeed, there was a strong negative correlation between the baseline anti-S1 IgG titer and the fold-change in anti-S1 IgG titers after first vaccination. Furthermore, differences between cohorts could arise from other differences prior to lymphocyte activation, such as during antigen-presenting cell priming, as the duration of the dysregulation of innate immune responses in the setting of COVID-19 remains unknown (*68*). Although there are diseases associated with the toxic accumulation of antibodies (such as light chain amyloidosis, nephrotoxicity due to multiple myeloma, and others), we are unaware of reports of these phenomena after immunization but may represent a theoretical risk. There are many other characteristics of the humoral response, such as neutralization capability, isotype, subclass, glycosylation pattern, and breadth of binding, which are likely to affect protection afforded by COVID-19 vaccines. In addition, it is unknown whether booster doses will lead to higher titers of antibodies or if they will only increase to some threshold, akin to minimal increase in titer that was observed with the second vaccination in SARS-CoV-2-experienced adults in our studies.

Although we observed differential induction of cellular and humoral responses to vaccination based on prior exposure to SARS-CoV-2, certain caveats apply to these studies. First, overall sample size was limited and thus our findings will need to be further validated in larger, diverse cohorts. Second, we tested BNT162b2 but were not able to test the many other COVID-19 vaccines being developed, nor did we assess later time points to determine longevity of the immune response. Furthermore, we opted to look one week after each vaccination based on prior influenza studies but cannot rule out that the timing of the peak cellular response occurred outside of the time points assessed. Future studies with blood draws shortly after vaccination, particularly after the second dose, may clarify whether a plasmablast response occurred. In addition, understanding the impact of pre-existing T and B cell memory due to seasonal coronaviruses on the magnitude of the COVID-19 vaccine response could improve the ability to predict the overall vaccine response. Finally, studies of the draining lymph node after vaccination may be needed to understand mechanisms underlying the differential responses observed here.

Together, these results highlight the importance of understanding prior immunological experience on the subsequent immune response to COVID-19 mRNA vaccines. Future studies will be needed to determine whether such personalized vaccination regimens will deliver durable, protective immunity to infection by the SARS-CoV-2 virus. Our results provide insight into the establishment and breadth of memory immune responses following mRNA vaccination.

## MATERIALS AND METHODS

### Study Design

We conducted an observational study of adults who were receiving BNT162b2 vaccination and willing to participate in the study, excluding individuals with severe anemia or inability to comply with study procedures. Thirty-six adults (21 SARS-CoV-2-naïve and 15 SARS-CoV-2-experienced) provided written consent for enrollment with approval from the NYU Institutional Review Board (protocols 18-02035 and 18-02037). Participants had blood drawn at defined time points, as diagrammed in **Fig. 1A**. Participant characteristics are summarized in **Tables S1** and **S2**. Participant-level data are presented in **data file S1**. Sample size calculations were not performed prior to the start of this non-randomized, non-interventional study. Outlier analyses were not performed.

### Blood samples processing and storage

Venous blood was collected by standard phlebotomy. Blood collection occurred at baseline, approximately one week after first vaccination (“Post 1st dose”), prior to the second vaccination (“Pre 2nd dose”), approximately one week after the second vaccination (“Post 2nd dose”), and one month after the second vaccination (“One month post 2nd dose”), as depicted in **Fig. 1A**. PBMCs were isolated from heparin vacutainers (BD Biosciences) that were stored overnight at room temperature, followed by processing using Sepmates (STEMCELL Technologies) in accordance with the manufacturer’s recommendations. Serum was collected in SST tubes (BD Biosciences) and frozen immediately at -80°C.

### Antibody ELISA and CXCL13 concentration evaluation

Direct ELISA was used to quantify antibody titers in participant serum. Ninety-six well plates were coated with 1 μg/mL S1 protein (100 μL/well) or 0.1 μg/mL N protein diluted in phosphate-buffered saline (PBS) and were then incubated overnight at 4°C (Sino Biological Inc., 40591-V08H and 40588-V08B). Plates were washed four times with 250 μL of PBS containing 0.05% Tween 20 (Thermo Fisher Scientific) (PBS-T) and blocked with 200 μl PBS-T containing 4% non-fat milk and 5% whey, as blocking buffer at room temperature for 1 hour. Serum samples were heated at 56°C for 1 hour prior to use. Samples were diluted to a starting concentration of 1:50 (S1), or 1:100 (N) were first added to the plates and then serially diluted 1:3 in blocking solution. The final volume in all wells after dilution was 100 μL. After a 2-hour incubation period at room temperature, plates were washed four times with PBS-T. Horseradish peroxidase (HRP)-conjugated goat-anti human IgG, IgM, and IgA (Southern BioTech, 2040-05, 2020-05, 2050-05) were diluted in blocking buffer (1:2000, 1:1000, 1:1000, respectively) and 100 μL was added to each well. Plates were incubated for 1 hour at room temperature and washed four times with PBS-T. After developing for 5 min with TMB Peroxidase Substrate 3,3′,5,5′-Tetramethylbenzidine (Thermo Fisher Scientific), the reaction was stopped with 1M sulfuric acid or 1N hydrochloric acid. The optical density was determined by measuring the absorbance at 450 nm on a Synergy 4 (BioTek) plate reader. To summarize data collected on individuals, the area under the response curve was calculated for each sample and end point titers were normalized using replicates of pooled positive control serum samples on each plate to reduce variability between plates. Measurement of CXCL13 concentrations was performed using plasma samples that had been stored at -80°C. Plasma was warmed to room temperature and immediately diluted 1:1 in buffer. Diluted plasma was placed into a CXCL13 Simple Plex Cartridge (ProteinSimple, SPCKB-PS-000375) according to the manufacturer’s instructions.

### Avidity assay

Ninety-six well plates were coated with 0.1 μg/mL S1 protein (100 μL/well) diluted in PBS overnight at 4°C (Sino Biological). Plates were washed four times with 250 μL of PBS containing 0.05% Tween 20 (PBS-T) and blocked with 200 μL PBS-T containing 4% non-fat milk and 5% whey, as blocking buffer at room temperature for 1 hour. Serum samples were heated at 56°C for 1 hour prior to use. Samples were diluted to a starting concentration of 1:50 and added to the plates in quadruplicate and then serially diluted 1:3 in blocking solution. The final volume in all wells after dilution was 100 μL. After a 2-hour incubation period at room temperature, plates were washed four times with PBS-T. PBS was then added to two dilution replicate sets and 6 M Urea to the other two dilution replicate sets. Plates were incubated for 10 min at room temperature before washing four times with PBS-T. Antibodies were detected and plates were developed and read as described above for ELISA assays. Avidity was calculated by dividing the dilutions that gave an optical density value of 0.5 (Urea treatment/no Urea). Scores with theoretical values between 0 and 100% were generated.

### Antibody-secreting cell ELISpot Assays

A direct enzyme-linked immunospot (ELISpot) assay was used to determine the number of SARS-CoV-2 spike protein subunit S1-, S2-, and RBD-specific IgG, IgA, and IgM ASCs in fresh PBMCs. Ninety-six well ELISpot filter plates (Millipore, MSHAN4B50) were coated overnight with 2 μg/mL recombinant S1, S2, or RBD (Sino Biological Inc., 40591-V08H, 40590-V08B, and 40592-V08H), or 10 μg/mL of goat anti-human IgG/A/M capture antibody (Jackson ImmunoResearch Laboratory Inc., 109-005-064). Plates were washed four times with 200 μL PBS-T and blocked for 2 hours at 37°C with 200 μL RPMI-1640 containing 10% fetal calf serum (FCS), 100 units/mL of penicillin G, and 100 μg/mL of streptomycin (Gibco), referred to as complete medium. Then 50 μL of cells in complete media at 10x10^6^ cells/mL were added to the top row of wells containing 150 μL complete media and 3-fold serial diluted three times. Plates were incubated overnight at 37°C with 5% CO_2_. Plates were washed once with 200 μL PBS followed by four times with 200 μL PBS containing 0.05% Tween 20 (PBS-T). Biotinylated anti-human IgG, IgM, or IgA antibody (Jackson ImmunoResearch Laboratory Inc., 709-065-098, 109-065-129, 109-065-011) were diluted 1:1000 in PBS-T with 2% FCS (Ab diluent) and 100 μL was added to wells for 2 hours at room temperature or overnight at 4°C. Plates were washed four times with 200 μL PBS-T and incubated with 100 μL of Avidin-D-HRP conjugate (Vector Laboratories, A-2004) diluted 1:1000 in PBS-T for 1 hour at room temperature. Plates were washed four times with 200 μL PBS-T and 100 mL of AEC substrate (3 amino-9 ethyl-carbazole; Sigma Aldrich, A-5754) was added. Plates were incubated at room temperature for five minutes and rinsed with water to stop the reaction. Developed plates were scanned and analyzed using an ImmunoSpot automated ELISpot counter (Cellular Technology Limited).

### SARS-CoV-2 microneutralization assay

Viral neutralization activity of plasma was measured in an immunofluorescence-based microneutralization assay by detecting the neutralization of infectious virus in cultured Vero E6 cells (African Green Monkey Kidney; ATCC #CRL-1586). These cells are known to be highly susceptible to infection by SARS-CoV-2. Cells were maintained according to standard ATCC protocols. Briefly, Vero E6 cells were grown in Dulbecco’s Modified Eagle Medium (DMEM) supplemented with 10% heat-inactivated fetal bovine serum (FBS), 2 mM L-glutamine, and 1% of MEM Nonessential Amino Acid (NEAA) Solution (Thermo Fisher Scientific, #MT25025CI). Cell cultures were grown in 75 or 150 cm^2^ flasks at 37°C with 5% CO_2_ and passaged 2 to 3 times per week using trypsin-EDTA. Cell cultures used for virus testing were prepared as subconfluent monolayers. All incubations containing cells were performed at 37°C with 5% CO_2_. All SARS-CoV-2 infection assays were performed in the CDC/USDA-approved BSL3 facility of NYU Grossman School of Medicine, in accordance with its Biosafety Manual and Standard Operating Procedures. SARS-CoV-2 isolate USA-WA1/2020, deposited by the Centers for Disease Control and Prevention, was obtained through Biodefense and Emerging Infections Research Resources Repository, National Institute of Allergy and Infectious Diseases, National Institutes of Health (NR-52281, GenBank accession no. MT233526). Serial dilutions of heat-inactivated plasma (56°C for 1 hour) were incubated with USA-WA1/2020 stock (at fixed 1x10^6^ PFU/mL) in DMEM supplemented with 2 mM L-glutamine, 1% of MEM Nonessential Amino Acid (NEAA) Solution, and 10 mM HEPES (Thermo Fisher Scientific 15-630-080) for 1 hour at 37°C. One hundred microliters of the plasma-virus mix was then added to the cells and incubated at 37°C with 5% CO_2_. Twenty-four hours post-infection, cells were fixed with 10% formalin solution (4% active formaldehyde) for 1 hour, stained with an α-SARS-CoV-2 nucleocapsid antibody at 1:2,000 (ProSci #10-605, RRID AB_2895130), and a goat α-mouse IgG alexa fluor 647 secondary antibody at 1:2,000 (Thermo Fisher Scientific A32728, RRID AB_2633277) along with 4′,6-diamidino-2-phenylindole (DAPI) at 1:4,000 and visualized by microscopy with the CellInsight CX7 High-Content Screening (HCS) Platform (Thermo Fisher Scientific) and high-content software (HCS) (*69*).

### Cellular phenotyping

Peripheral blood was collected in sodium heparin collection tubes and maintained at room temperature overnight. PBMCs were isolated using the Sepmate system (STEMCELL Technologies) in accordance with manufacturer’s instructions. Then, 2 to 5 million freshly isolated PBMCs were resuspended in HBSS supplemented with 1% FCS (Thermo Fisher Scientific) and 0.02% sodium azide (Sigma-Aldrich). Cells underwent Fc-blockade with Human TruStain FcX (BioLegend) and NovaBlock (Thermo Fisher Scientific) for 10 min at room temperature, followed by surface staining antibody cocktail at room temperature for 20 min in the dark. Cells were permeabilized with the eBioscience Intracellular Fixation and Permeabilization kit (Thermo Fisher Scientific) for 20 min at room temperature in the dark, followed by intracellular staining with an antibody cocktail for 1 hour at room temperature in the dark. All samples were then resuspended in 1% paraformaldehyde and acquired within three days of staining on a 5-laser Aurora cytometer (Cytek Biosciences). Antibodies, clones, and catalog numbers are described in **table S3**. Initial data quality control was performed using FlowJo (BD Biosciences). Non-naive CD8^+^ and CD4^+^ T cells were analyzed in the OMIQ.ai platform (www.omiq.ai) using Phenograph clustering (*32*) with k=20 and a Euclidean distance metric, followed by tSNE projection. Heatmaps and differential cluster abundance were assessed by edgeR (*70*) using OMIQ.ai.

### Activation-induced marker analysis

Cryopreserved PBMCs were thawed and rested overnight at 37°C in RPMI-1640 with L-glutamine (Thermo Fisher Scientific) containing 10% FCS (Thermo Fisher Scientific), 2 mM L-glutamine (Thermo Fisher Scientific), and 100 U/ml of penicillin-streptomycin (Thermo Fisher Scientific). The following day, cells were stimulated with 0.6 nmol of each of the S1, S, and S+ PepTivator pools (Miltenyi) for 20 hours at 37°C with 1.5x10^6^ cells per well in a 96 well flat bottom plate. For the unstimulated control well, sterile water was used in place of the peptide pools. Monensin (Thermo Fisher Scientific) was added for the last 6 hours of stimulation at a final concentration of 10 μM. After stimulation, cells were washed with PBS containing 10 mM EDTA at 37°C for 5 min, followed by Fc-blockade and were stained as previously described. Antibodies, clones, and catalog numbers are described in **table S3**. Analysis was performed using FlowJo.

### B cell tetramer assays

Recombinant biotinylated RBD (BioLegend) was reacted with PE-Streptavidin (BioLegend) in a 4:1 molar ratio at 4°C for 2 hours. For flow cytometry studies, PBMCs underwent Fc-blockade with Human TruStain FcX (BioLegend), NovaBlock (Thermo Fisher Scientific), APC-Streptavidin (BioLegend), and mouse phycoerythrin (PE)-IgG2b isotype control (BioLegend) for 10 min at room temperature, followed by allophycocyanin (APC) anti-PE (BioLegend) for 10 min. Then cells were washed twice and stained with the RBD tetramer and antibodies against other surface proteins for 20 min at room temperature in the dark, followed by resuspension in 1% paraformaldehyde and acquisition on a 5-laser Aurora cytometer (Cytek Biosciences).

### Statistical analysis

Primary data analysis and statistical analysis were performed using the R environment (version 4.0.2). Bioinformatics analysis scripts are available online at Zenodo (doi: 10.5281/zenodo.5731495). All experiments were performed once using the number of biological replicates indicated in the figure legends. Statistical tests were performed using the “rstatix” library (version 0.6.0) and conducted as two-tailed tests with α=0.05. Nonparametric 2-sample Wilcoxon tests were performed preferentially throughout. Non-parametric Kruskal-Wallis test was used with Dunn’s post-test in settings with 3 or more comparisons. Correlation analyses were performed as nonparametric tests using Kendall’s tau statistic. Study schematics were made with BioRender.

## Supplementary Materials

### This PDF file includes:

Figs. S1 to S6 Tables S1 to S3 Reference (*71*)

### Other Supplementary Material for this manuscript includes the following:

Data file S1

MDAR Reproducibility Checklist
