# Supplementary material for: Robust immune responses are observed after one dose of BNT162b2 mRNA vaccine dose in SARS-CoV-2 experienced individuals
Source: Sci Transl Med. 2021 Dec 7:eabi8961. doi: 10.1126/scitranslmed.abi8961 (PMC9248013; doi:10.1126/scitranslmed.abi8961)
Supplement: Supplementary file 1 — Figs. S1 to S6 Tables S1 to S3 Reference (71) [file scitranslmed.abi8961_sm.pdf]

Science Translational Medicine
AAAS

Supplementary Materials for

Robust immune responses are observed after one dose of BNT162b2 mRNA vaccine dose in SARS-CoV-2 experienced individuals

Marie I. Samanovic *et al.*

Corresponding authors: Ramin Sedaghat Herati, ramin.herati@nyulangone.org;
Mark J. Mulligan, mark.mulligan@nyulangone.org

DOI: 10.1126/scitranslmed.abi8961

The PDF file includes:

Fig. S1 to S7
Tables S1 to S3
Reference (*71*)

Other Supplementary Material for this manuscript includes the following:

Data file S1
MDAR Reproducibility Checklist

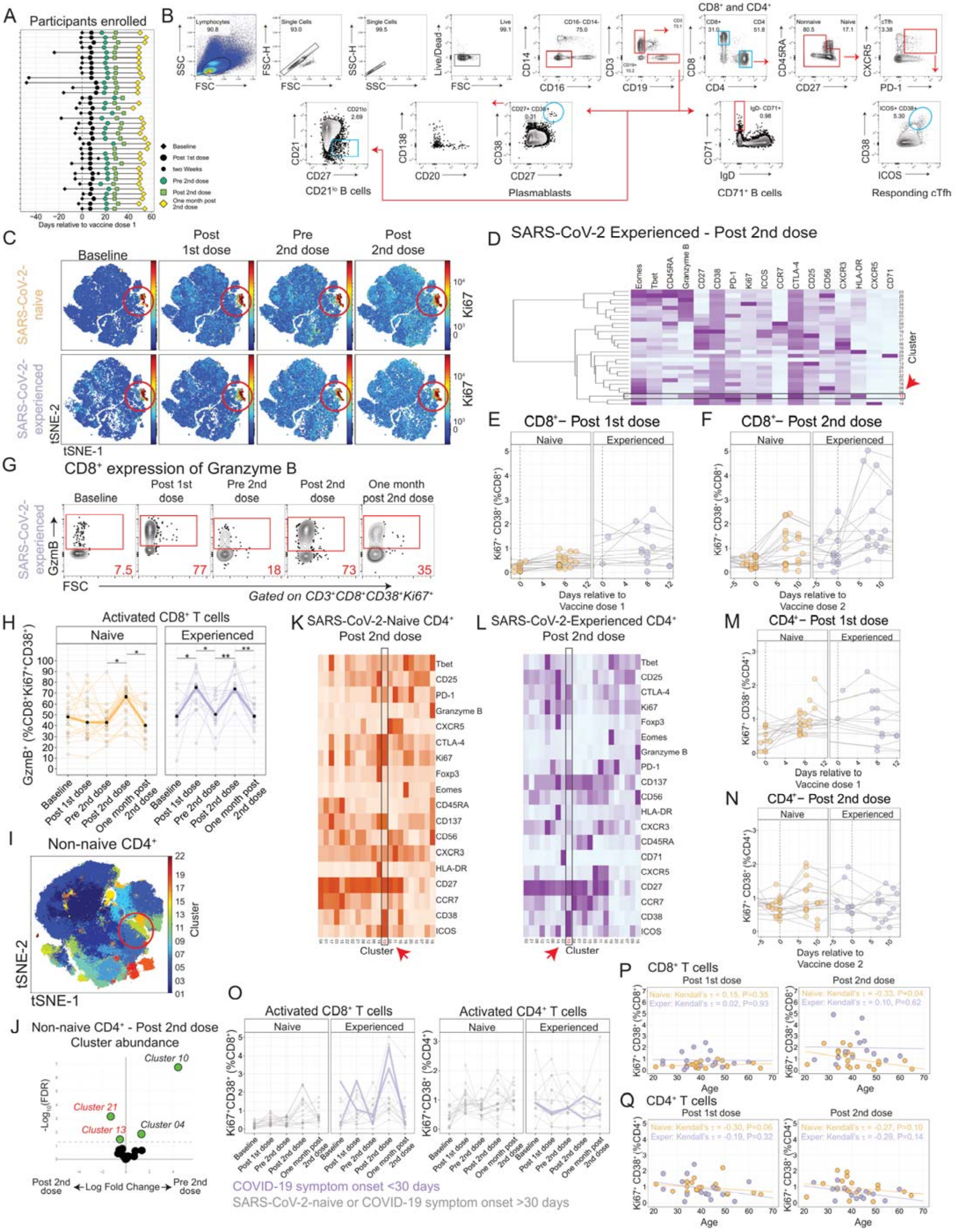
A
Participants enrolled
Baseline
Post 1st dose
two Weeks
Pre 2nd dose
Post 2nd dose
One month post 2nd dose
Days relative to vaccine dose 1
B
CD8+ and CD4+
Lymphocytes
Single Cells
Live
CD16- CD14-
CD8+
CD4
Nonnaive
Naive
cTfh
CD21lo
CD27+ CD38+
IgD- CD71+
ICOS+ CD38+
CD21lo B cells
Plasmablasts
CD71+ B cells
Responding cTfh
C
Baseline
Post 1st dose
Pre 2nd dose
Post 2nd dose
SARS-CoV-2-naive
SARS-CoV-2-experienced
Ki67
tSNE-2
tSNE-1
D
SARS-CoV-2 Experienced - Post 2nd dose
Eomes
Tbet
CD45RA
Granzyme B
CD27
CD38
PD-1
Ki67
ICOS
CCR7
CTLA-4
CD25
CD56
CXCR3
HLA-DR
CXCR5
CD71
Cluster
E
CD8+– Post 1st dose
Naive
Experienced
Ki67+ CD38+ (%CD8+)
Days relative to Vaccine dose 1
F
CD8+– Post 2nd dose
Naive
Experienced
Days relative to Vaccine dose 2
G
CD8+ expression of Granzyme B
Baseline
Post 1st dose
Pre 2nd dose
Post 2nd dose
One month post 2nd dose
SARS-CoV-2-experienced
GzmB
FSC
7.5
77
18
73
35
Gated on CD3+CD8+CD38+Ki67+
H
Activated CD8+ T cells
Naive
Experienced
GzmB+ (%CD8+Ki67+CD38+)
I
Non-naive CD4+
Cluster
tSNE-2
tSNE-1
J
Non-naive CD4+ - Post 2nd dose
Cluster abundance
Cluster 10
Cluster 21
Cluster 04
Cluster 13
-Log10(FDR)
Post 2nd dose
Log Fold Change
Pre 2nd dose
K
SARS-CoV-2-Naive CD4+
Post 2nd dose
Cluster
L
SARS-CoV-2-Experienced CD4+
Post 2nd dose
Cluster
M
CD4+– Post 1st dose
Naive
Experienced
Ki67+ CD38+ (%CD4+)
Days relative to Vaccine dose 1
N
CD4+– Post 2nd dose
Naive
Experienced
Days relative to Vaccine dose 2
O
Activated CD8+ T cells
Activated CD4+ T cells
Naive
Experienced
Ki67+CD38+ (%CD8+)
Ki67+CD38+ (%CD4+)
COVID-19 symptom onset <30 days
SARS-CoV-2-naive or COVID-19 symptom onset >30 days
P
CD8+ T cells
Post 1st dose
Post 2nd dose
Naive: Kendall's τ = 0.15, P=0.35
Exper: Kendall's τ = 0.02, P=0.93
Naive: Kendall's τ = -0.33, P=0.04
Exper: Kendall's τ = 0.10, P=0.62
Age
Q
CD4+ T cells
Post 1st dose
Post 2nd dose
Naive: Kendall's τ = -0.30, P=0.06
Exper: Kendall's τ = -0.19, P=0.32
Naive: Kendall's τ = -0.27, P=0.10
Exper: Kendall's τ = -0.29, P=0.14
Age

Fig. S1. $CD4^+$ and $CD8^+$ T cell responses and gating strategy.

(**A**) Study participant timeline is shown relative to first vaccination. (**B**) The gating scheme for T and B cell populations is shown. FSC (forward scatter) and SSC (side-scatter) channels were used to identify lymphocytes, and FSC-Height (FSC-H) and SSC-Height (SSC-H) were used to identify single cells. Responding circulating T follicular helper cells (cTfh) were identified based on expression of CXCR5 and PD-1. (**C**) The non-naive $CD8^+$ T cell population is shown in t-Stochastic Neighbor Embedding (tSNE) projection for SARS-CoV-2-naive (upper) or SARS-CoV-2-experienced (lower) participants. Heatmaps shows expression of Ki67. Circled areas indicate the region corresponding to Cluster 12. (**D**) Non-naive $CD8^+$ T cells underwent Phenograph clustering. Protein expression for each cluster for SARS-CoV-2-experienced adults are shown at one week after second dose. Red arrow indicates Cluster 12. (**E** and **F**) $Ki67^+CD38^+$ expression in $CD8^+$ T cells is shown by cohort over time measured in days, relative to the individual's first (E) or second (F) vaccination for SARS-CoV-2-naive (orange, n=19) and SARS-CoV-2-experienced (purple, n=14) participants. (**G**) Representative flow cytometry plots are shown for $Ki67^+CD38^+$ $CD8^+$ T cell expression of granzyme B (GzmB) in a SARS-CoV-2-experienced individual. Red numbers indicate frequency. (**H**) Summary data for $Ki67^+CD38^+$ $CD8^+$ T cell expression of granzyme B for SARS-CoV-2-naive (orange, N=21) and SARS-CoV-2-experienced (purple, N=14) participants. * $P<0.05$ and ** $P<0.01$ by Dunn's post-test. (**I**) Non-naive $CD4^+$ T cells from all samples were merged for tSNE projection. Colors indicate Phenograph clustering. (**J**) Phenograph cluster abundance for non-naive $CD4^+$ T cells is shown for all participants before and after second vaccination. (**K and L**) Protein expression is shown for Phenograph clusters for non-naive $CD4^+$ T cells shown for samples at one week following second vaccination in SARS-CoV-2-naive (K) or SARS-CoV-2-experienced (L) participants. (**M and N**) $CD4^+$ T cells were evaluated for expression of Ki67 and CD38 after vaccination over time measured in days, relative to the individual's first (M) or second (N) vaccinations for SARS-CoV-2-naive (orange, n=19) and SARS-CoV-2-experienced (purple, n=14) participants. (**O**) Summary data for $Ki67^+CD38^+$ expression on $CD8^+$ T cells (left) and $CD4^+$ T cells (right) is shown by cohort highlighting the two participants with recent COVID-19 in purple. (**P and Q**) Kendall correlation is shown between $Ki67^+CD38^+$ expression in $CD4^+$ (P) or $CD8^+$ (Q) T cells and age one week after either first vaccination (left) or second vaccination (right) for SARS-CoV-2-naive (orange, n=21) and SARS-CoV-2-experienced (purple, n=14) participants.

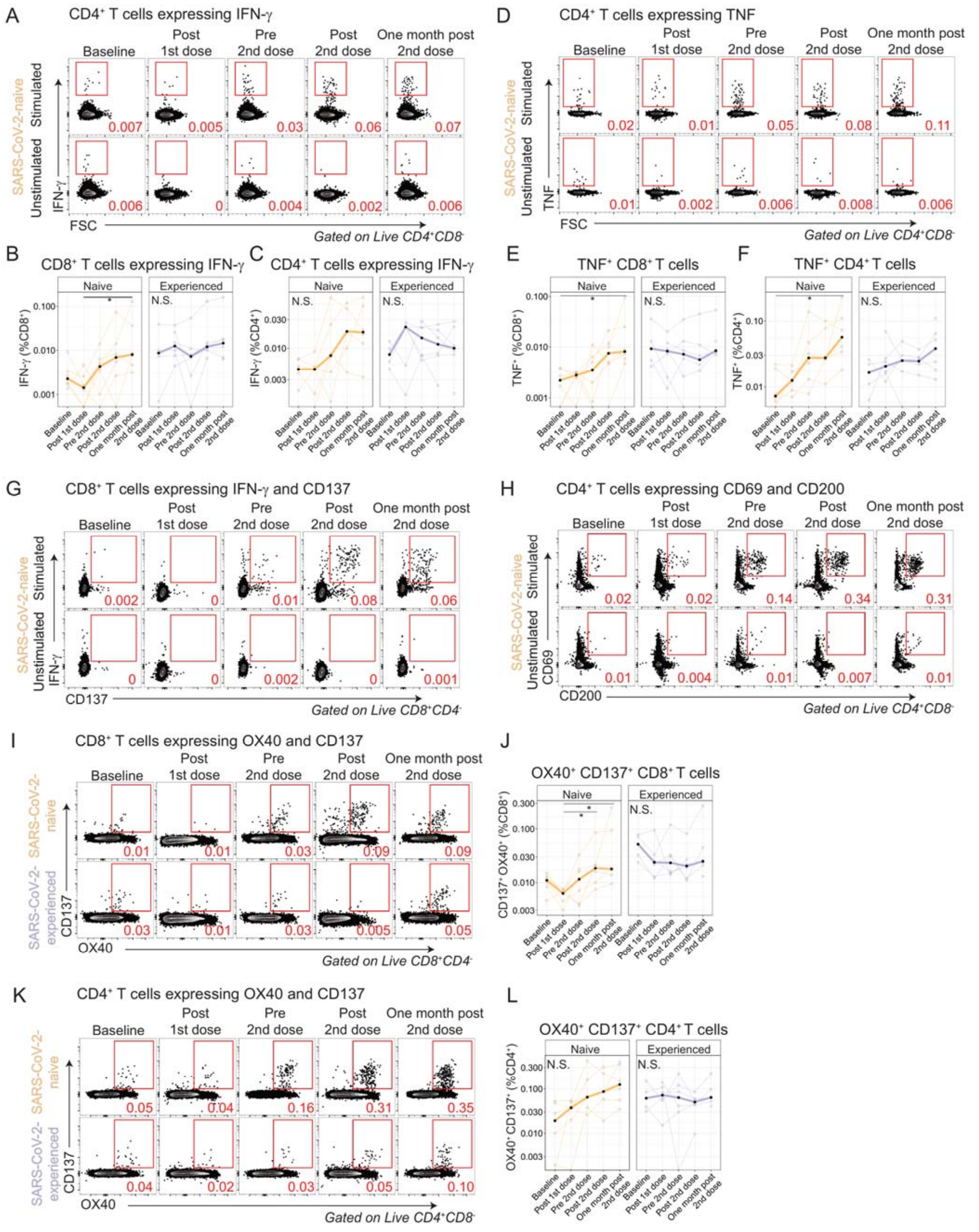

Fig. S2. Antigen-specific T cell responses to vaccination.

Peripheral blood mononuclear cells were rested overnight, then stimulated for 20 hours with SARS-CoV-2 spike peptides in the presence of monensin, followed by phenotyping for flow cytometry. For all panels, **P* < 0.05 by Dunn's post-test. (**A**) $CD4^+$ T cells evaluated for expression of interferon (IFN)-γ are shown in a SARS-CoV-2-naive individual with peptide stimulation (top row) or no stimulation (bottom row). Red numbers indicate frequency. (**B and C**) Summary plots are shown for expression of IFN-γ in $CD8^+$ (B) or $CD4^+$ (C) T cells for SARS-CoV-2-naive (orange, n=6) and SARS-CoV-2-experienced (purple, n=6) participants. (**D**) Representative flow cytometry plots are shown for $CD4^+$ T cells evaluated for expression of tumor necrosis factor (TNF) in a SARS-CoV-2-naive individual with peptide stimulation (top row) or no stimulation (bottom row). Red numbers indicate frequency. (**E and F**) Summary plots are shown for expression of TNF in $CD8^+$ (E) or $CD4^+$ (F) T cells for SARS-CoV-2-naive (orange, n=6) and SARS-CoV-2-experienced (purple, n=6) participants. (**G**) Representative flow cytometry plots show $CD8^+$ T cell expression of IFN-γ and CD137 in samples from one SARS-CoV-2-naive individual across all time points for stimulated (top row) or unstimulated (bottom row) conditions. Red numbers indicate frequency. (**H**) Representative flow cytometry plots show $CD4^+$ T cell expression of CD69 and CD200 for stimulated and unstimulated conditions. Red numbers indicate frequency. (**I to L**) Expression of OX40 and CD137 in $CD8^+$ (I) or $CD4^+$ (K) T cells was measured in samples from SARS-CoV-2-naive (top row) and SARS-CoV-2-experienced (bottom row) individuals, with summary plots shown for $CD8^+$ (J) and $CD4^+$ (L) at right for SARS-CoV-2-naive (orange, n=6) and SARS-CoV-2-experienced (purple, n=6) participants. Red numbers indicate frequency.

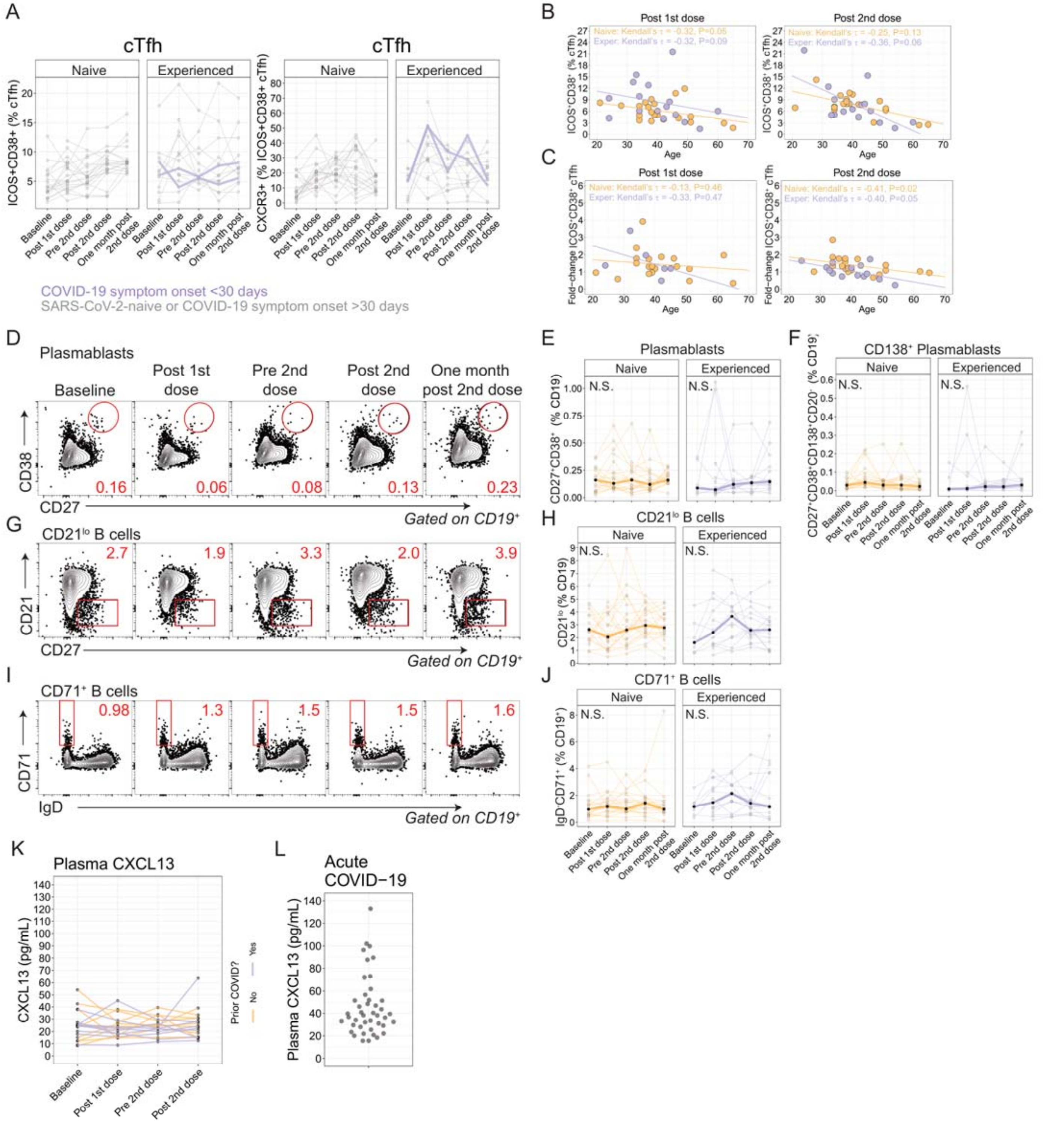

Fig. S3. Plasmablast and CXCL13 responses to vaccinations.

(**A**) Summary data is shown for expression of Inducible Costimulator (ICOS) and CD38 in cTfh (left) and CXCR3 expression in ICOS$^+$CD38$^+$ cTfh (right) by cohort, with purple lines indicating the two participants with recent COVID-19. (**B**) cTfh expressing ICOS and CD38 were negatively correlated with age one week after the first vaccination (left) or the second vaccination (right) for SARS-CoV-2-naive (orange, n=21) and SARS-CoV-2-experienced (purple, n=14) participants. (**C**) The fold-change in cTfh expressing ICOS and CD38 at one week after the first vaccination compared to baseline (left) or 1 week after the second vaccination compared to Pre 2nd dose (right) was negatively correlated

with age for SARS-CoV-2-naive (orange, n=21) and SARS-CoV-2-experienced (purple, n=14) participants. (**D**) Plasmablasts were identified by expression of CD27 and CD38. An example plot is shown. Red numbers indicate frequency. (**E**) Summary data for plasmablasts identified by high expression of CD27 and CD38 is shown for samples isolated from SARS-CoV-2-naive (orange, n=21) and SARS-CoV-2-experienced (purple, n=14) participants. (**F**) Summary data is shown for plasma cells, defined as $CD27^{+}CD38^{hi}CD138^{+}CD20^{-}$, as a proportion of $CD19^{+}$ B cells isolated from SARS-CoV-2-naive (orange, n=21) and SARS-CoV-2-experienced (purple, n=14) participants. (**G**) Example plots show gating for $CD21^{lo}$ B cells. Red numbers indicate frequency. (**H**) Summary data for $CD21^{lo}$ B cells is shown longitudinally for samples isolated from SARS-CoV-2-naive (orange, n=21) and SARS-CoV-2-experienced (purple, n=14) participants. (**I**) Example plots show gating for $CD71^{+}$ B cells. Red numbers indicate frequency. (**J**) Summary data are shown for $CD71^{+}$ B cells longitudinally isolated from SARS-CoV-2-naive (orange, n=21) and SARS-CoV-2-experienced (purple, n=14) participants. (**K**) Plasma CXCL13 was measured longitudinally for both SARS-CoV-2-naive (orange, n=9) and SARS-CoV-2-experienced (purple, n=9) participants. (**L**) Plasma CXCL13 is shown for an independent cohort of patients with acute COVID-19 who were sampled within 30 days of the onset of symptoms (n=43).

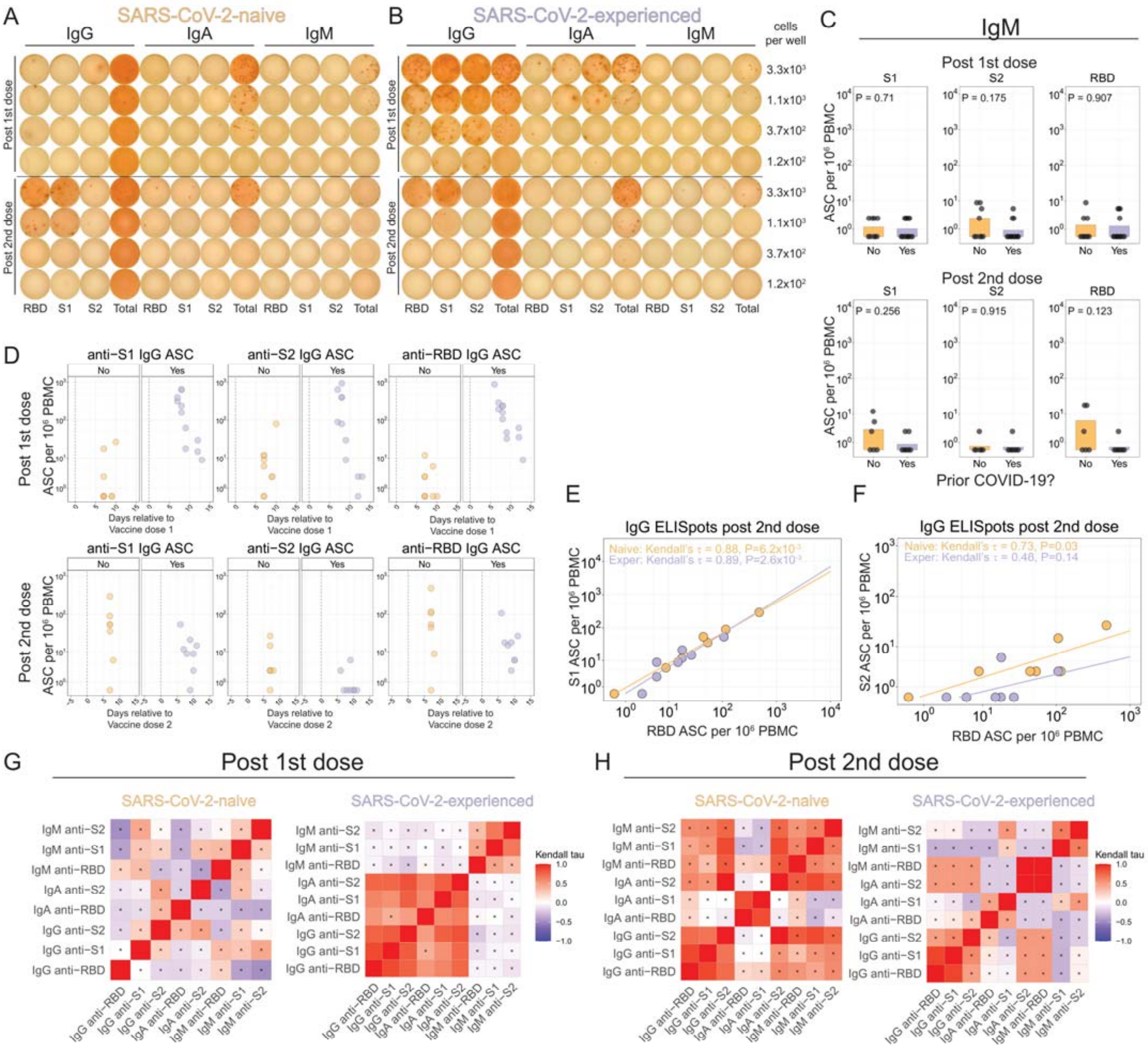

Fig. S4. Muted IgG and IgA ASC responses to second dose in SARS-CoV-2 experienced participants.

(**A and B**) Antibody-secreting cell (ASC) ELISpots are shown for IgG, IgA, and IgM-producing cells reacting to RBD, S1, or S2 antigens, or total secreted antibody controls. (**C**) IgM-producing ASCs in circulation were quantified one week after first vaccination (top) for SARS-CoV-2-naive (orange, n=9) and SARS-CoV-2-experienced (purple, n=13) participants or one week after second vaccination (bottom) for SARS-CoV-2-naive (n=6) and SARS-CoV-2-experienced (n=8) participants. Nominal *P* values from Wilcoxon tests are presented. (**D**) Frequencies of ASCs specific to S1, S2, or RBD antigens were measured in days relative to the first vaccination (top) for SARS-CoV-2-naive (orange, n=9) and SARS-CoV-2-experienced (purple, n=12) participants or second vaccination (bottom) for SARS-CoV-2-naive (n=7) and SARS-CoV-2-experienced (n=8) participants. (**E and F**) Pearson correlations are shown for S1-reactive IgG ASCs (E) for SARS-CoV-2-naive (orange, n=6) and SARS-CoV-2-experienced (purple, n=8) participants or S2-reactive IgG ASCs (F) compared to RBD-reactive IgG ASCs one week after second vaccination for SARS-CoV-2-naive (n=7) and SARS-CoV-

2-experienced (n=7) participants. (**G and H**) Kendall correlations are shown for SARS-CoV-2-specific frequencies for SARS-CoV-2-naive or SARS-CoV-2-experienced adults one week after first (G) or one week after second (H) vaccination for SARS-CoV-2-naive (n=9) and SARS-CoV-2-experienced (n=13) participants. Heatmaps are colored by Kendall's tau statistic. Boxes with symbols indicate nominal *P* value >0.05.

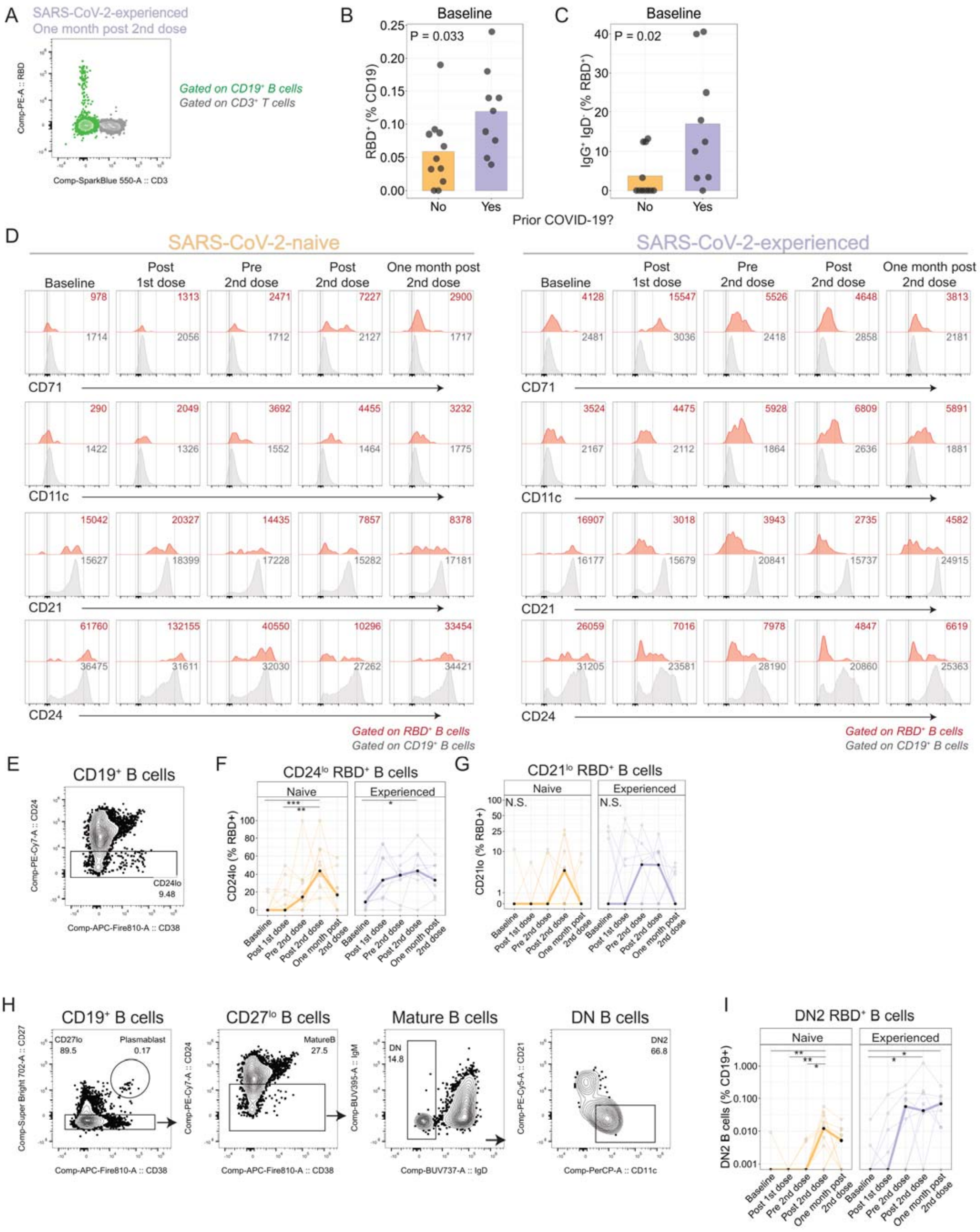

Fig. S5. RBD-reactive B cells following immunization.

(**A**) Recombinant biotinylated RBD was tetramerized using phycoerythrin (PE)-streptavidin. A SARS-CoV-2-experienced individual late after vaccination was used as an initial positive control. Plot shows $CD19^+$ B cells (green) or $CD3^+$ T cells (gray) for RBD binding. (**B**) RBD-reactive B cells were compared at baseline for SARS-CoV-2-naive (orange, n=11) or SARS-CoV-2-experienced (purple, n=9) participants (*P*=0.03, Wilcoxon test). (**C**) Proportion of IgG-expressing B cells among all RBD^+ B cells is shown by cohort for SARS-CoV-2-naive (orange, n=11) or SARS-CoV-2-experienced (purple, n=9) participants (*P*=0.02, Wilcoxon test). (**D**) B cell phenotypic analysis is shown. Red histogram depicts RBD^+ B cells and the gray histogram depicts all $CD19^+$ B cells, with the numbers indicating the mean fluorescence intensity for the respective populations for the proteins shown in each row. (**E**) The gating scheme for identification of $CD24^{lo}$ B cells is shown. (**F and G**) Summary plots for $CD21^{lo}$ B cells (F) and $CD24^{lo}$ B cells (G) among all RBD^+ B cells are shown for samples isolated from SARS-CoV-2-naive (n=9) and SARS-CoV-2-experienced (n=11) participants. (**H**) The gating scheme for identification of double-negative 2 (DN2) B cells is shown. (**I**) Summary plots for proportions of DN2 B cells are shown for samples isolated form SARS-CoV-2-naive (n=9) and SARS-CoV-2-experienced (n=11) participants. * $P < 0.05$, ** $P < 0.01$, and ***$P < 0.001$ by Dunn's post-test.

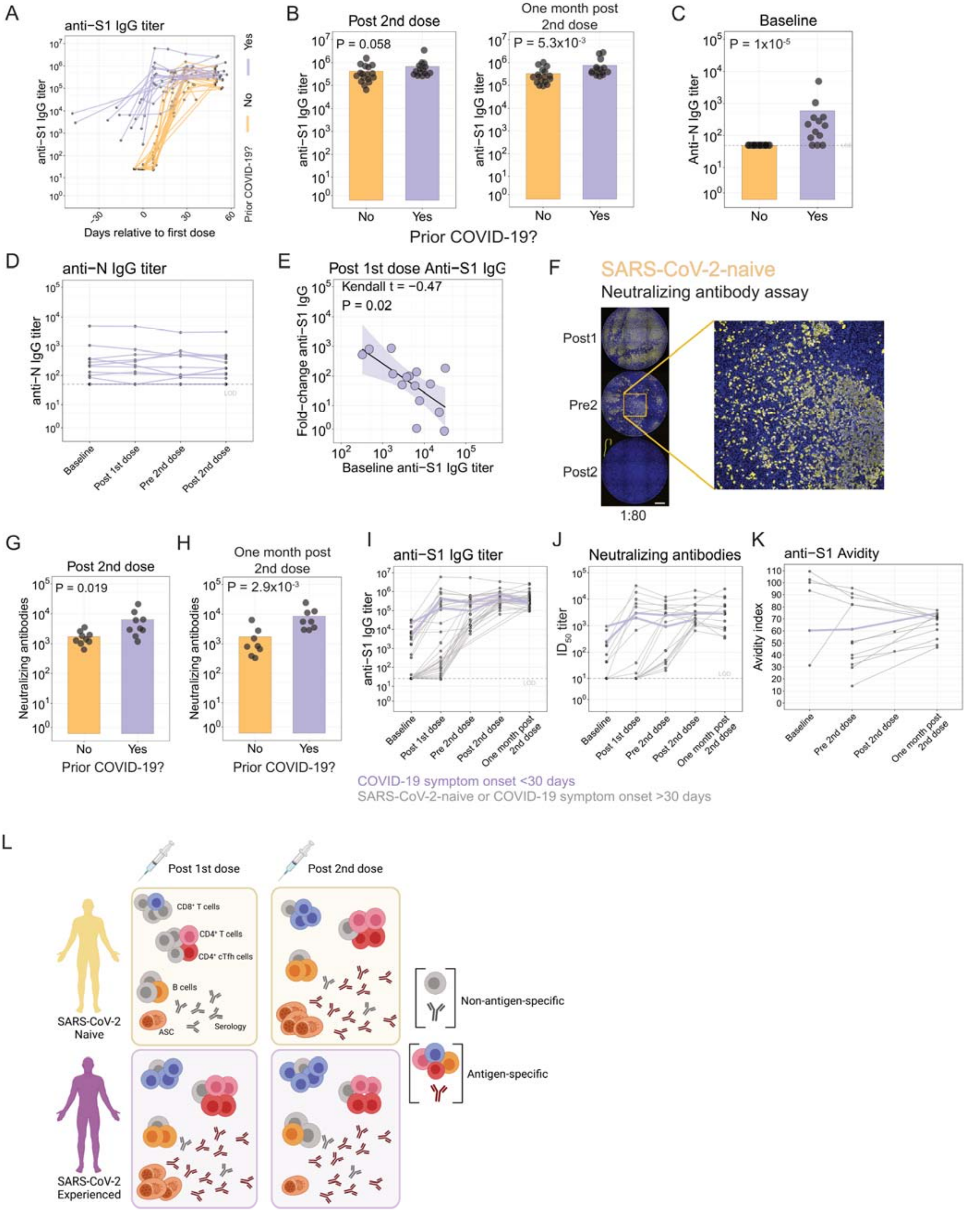

Fig. S6. Robust anti-S1 binding and neutralizing antibody responses are observed in SARS-CoV-2-experienced individuals after vaccination.

(**A**) Anti-S1 IgG serum antibody titers were measured over time in days relative to the first vaccination for SARS-CoV-2-naive (orange, n=21) and SARS-CoV-2-experienced (purple, n=15) participants. (**B**) Anti-S1 IgG titers are shown at Post 2nd dose (left) or at the One month post 2nd dose time point (right) for SARS-CoV-2-naive (n=16) and SARS-CoV-2-experienced (n=15) participants. (**C**) Anti-nucleocapsid IgG serum antibody titers are shown for SARS-CoV-2-naive (n=19) and SARS-CoV-2-experienced (n=13) participants. (**D**) Anti-nucleocapsid IgG serum antibody titers are shown for SARS-CoV-2-experienced participants over time (n=13). (**E**) The correlation between fold-change in anti-S1 IgG serum antibody titers, assessed at one week after vaccination compared to Baseline, is shown for SARS-CoV-2-experienced participants (n=15). (**F**) An example of the neutralizing antibody assay is shown for the same SARS-CoV-2-naive participant longitudinally at 1:80 plasma dilution (left), with magnification of well image (right). Scale bar indicates 100 μm. (**G**) Neutralizing antibody titers are shown Post 2nd dose for SARS-CoV-2-naive (orange, n=9) and SARS-CoV-2-experienced (purple, n=9) participants (P=0.02; Wilcoxon test). (**H**) Neutralizing antibody titers one month after the 2nd dose are shown for SARS-CoV-2-naive (orange, n=9) and SARS-CoV-2-experienced (purple, n=9) participants ($P=2.9\times10^{-3}$; Wilcoxon test). (**I to K**) Summary graphs of the anti-S1 IgG antibody titers (I), the inhibitory dilution (ID_{50}) neutralizing antibody titers (J), anti-S1 IgG antibody avidity (K) are shown for both cohorts, indicating participants with recent COVID-19 (purple). (**L**) Model for antigen-specific responses for SARS-CoV-2-naive and SARS-CoV-2-experienced individuals post first and second dose.

Table S1. Participant demographics.

	SARS-CoV-2-naive	SARS-CoV-2-experienced
**Number of participants**	21	15
**Age**		
**Median**	39	43
**Range**	21 to 65	24 to 60
**Sex (% Female)**	48%	67%
**Race (%)**		
**White or Caucasian**	76%	87%
**Asian**	19%	13%
**Black or African-American**	5 %	0 %
**Days between diagnosis of COVID-19 and first dose of vaccine**		
**Median**		282
**Range**		20 to 359

Table S2. Clinical COVID-19 disease among SARS-CoV-2-experienced participants.

Participant	Number of days between onset of COVID-19 symptoms and first dose of Vaccine	COVID-19 diagnostic test	World Health Organization COVID-19 Severity Score *(71)*	Days Hospitalized	Outcome
CV-043	360	NAAT$	7	17	Required intubation, treated with immunomodulator, improved and was discharged from hospital
CV-025	358*	anti-S1 IgG ELISA	1	0	Asymptomatic
CV-039	323	NAAT	2	0	Convalescent
CV-010	301	Commercial antibody test	2	0	Convalescent
CV-028	291	anti-S1 IgG ELISA	2	0	Convalescent
CV-034	290	NAAT	2	0	Convalescent
CV-015	285	NAAT	2	0	Convalescent
CV-005	284	Commercial antibody test	2	0	Convalescent
CV-020	282	NAAT	2	0	Convalescent
CV-027	281	NAAT	2	0	Convalescent
CV-026	279	NAAT	2	0	Convalescent
CV-033	275	anti-S1 IgG ELISA	1	0	Asymptomatic
CV-014	267	NAAT	2	0	Convalescent
CV-018	28	NAAT	2	0	Convalescent
CV-016	21	NAAT	2	0	Convalescent

*Number of days between diagnosis of asymptomatic COVID-19 and first dose of vaccine.
$NAAT, nucleic acid amplification test

Table S3. Antibodies used for flow cytometry experiments.

Target	Fluorochrome	Dilution	Clone	Manufacturer	Catalog #	RRID
**Live/Dead Blue**	-	-	-	Invitrogen	L23105	
**CD3**	APC/Fire 810	1:1000	SK7	BioLegend	344857	AB_2860894
**CD4**	SparkBlue 550	1:500	SK3	BioLegend	344656	AB_2819979
**CD4**	SparkViolet 538	1:300	SK3	BioLegend	344674	AB_2890774
**CD8**	PE-Fire 640	1:1000	SK1	BioLegend	344761	AB_2860887
**CD8**	SparkBlue 550	1:500	SK1	BioLegend	344759	AB_2819982
**CD11c**	PerCP	1:50	Bu15	BioLegend	337234	AB_2566656
**CD14**	BUV805	1:100	M5E2	BD Biosciences	612902	AB_2870189
**CD16**	BV480	1:500	3G8	BD Biosciences	566171	AB_2739568
**CD19**	BUV496	1:100	SJ25C1	BD Biosciences	612939	AB_2870221
**CD20**	APC	1:100	2H7	BioLegend	302309	AB_314257
**CD21**	PE-Cy5	1:200	B-ly4	BD Biosciences	551064	AB_394028
**CD23**	BUV615	1:1000	M-L233	BD Biosciences	751104	AB_2875134
**CD24**	PE-Cy7	1:100	ML5	BioLegend	311120	AB_2259843
**CD25**	BUV563	1:50	2A3	BD Biosciences	612919	AB_2870204
**CD27**	SB702	1:100	O323	Invitrogen	67-0279-42	AB_2762570
**CD38**	Qdot655	1:100	HIT2	Invitrogen	Q22150	AB_2556506
**CD38**	APC/Fire810	1:500	HIT2	BioLegend	303549	AB_2860783
**CD40**	BV510	1:100	5C3	BioLegend	334330	AB_2564034
**CD45RA**	Spark NIR 685	1:200	HI100	BioLegend	304168	AB_2832590
**CD56**	BV570	1:50	5.1H11	BioLegend	362539	AB_2565917
**CD69**	PE-Dazzle	1:100	FN50	BioLegend	310942	AB_2564277
**CD71**	SB780	1:50	OKT9	Invitrogen	78-0719-42	AB_2784898
**CD123**	BV650	1:50	7G3	BD Biosciences	563405	AB_2738185
**CD134 (OX40)**	BV421	1:100	ACT35	BioLegend	350014	AB_2564184
**CD137 (41BB)**	PE	1:100	4B4-1	BioLegend	309803	AB_314782
**CD137**	BV750	1:100	4B4-1	BD Biosciences	747353	AB_2872051
**CD138**	PacBlue	1:100	MI15	BioLegend	356531	AB_2687072
**CD150 (CTLA4)**	BV421	1:50	BNI3	BioLegend	369606	AB_2616795
**CD183 (CXCR3)**	BV750	1:100	1C6	BD Biosciences	746895	AB_2871692
**CD185 (CXCR5)**	BB515	1:200	RF8B2	BD Biosciences	564624	AB_2738871
**CD197 (CCR7)**	BV605	1:50	G043H7	BioLegend	353224	AB_2561753
**CD200 (OX2)**	PE-Cy7	1:200	OX-104	BioLegend	329211	AB_2563246
**CD278 (ICOS)**	APC/Fire750	1:100	C398.4A	BioLegend	313536	AB_2632923
**CD279 (PD-1)**	BB700	1:50	EH12.1	BD Biosciences	566460	AB_2744348
**HLA-DR**	BUV661	1:1000	G46-6	BD Biosciences	612980	AB_2870252
**IgM**	BUV395	1:200	G20-127	BD Biosciences	563903	AB_2721269
**IgD**	BUV737	1:500	IA6-2	BD Biosciences	612798	AB_2870125
**Foxp3**	PE-Cy5.5	1:50	PCH101	Invitrogen	35-4776-42	AB_11218682

**Tbet**	PE-Cy7	1:200	4B10	BioLegend	644823	AB_2561760
**Eomes**	PE-eF610	1:50	WD1928	Invitrogen	61-4877-42	AB_2574616
**GzmB**	A700	1:500	GB11	BD Biosciences	561016	AB_2033973
**Ki67**	BUV395	1:50	B56	BD Biosciences	564071	AB_2738577
**IgG**	PerCP-Vio700	1:100	IS11-3B2.2.3	Miltenyi	130-119-880	AB_2784374
**TNF**	BUV396	1:100	MAb11	BD Biosciences	563996	AB_2738533
**IFN-γ**	BV480	1:200	B27	BD Biosciences	566176	AB_2739573
